# IL-1β stimulated human umbilical cord mesenchymal stem cells ameliorate rheumatoid arthritis via inducing apoptosis of fibroblast-like synoviocytes

**DOI:** 10.1038/s41598-023-42585-1

**Published:** 2023-09-15

**Authors:** Yun-Hsuan Chiu, Ya-Han Liang, Jeng-Jong Hwang, Hwai-Shi Wang

**Affiliations:** 1 Institute of Anatomy and Cell Biology, School of Medicine, National Yang Ming Chiao Tung University, Peitou, Taipei, 112 Taiwan, ROC; 2 Department of Medical Imaging, Chung Shan Medical University Hospital affiliated with Medical Imaging and Radiological Sciences, Chung Shan Medical University, Taichung, 402 Taiwan, ROC

**Keywords:** Cell biology, Stem cells

## Abstract

Rheumatoid arthritis (RA) is characterized by synovial proliferation and lymphocyte accumulation leading to progressive damage of the periarticular bone and the articular cartilage. The hyperplasia of the synovial intima lining mainly consists of fibroblast-like synoviocytes-rheumatoid arthritis (HFLS-RA) which exhibit apoptosis-resistance, hyper-proliferation, and high invasiveness. The therapeutic efficacy of mesenchymal stem cells (MSCs) treatment in RA has been shown to be due to its immuno-regulatory ability. However, the exact factors and mechanisms involved in MSCs treatment in RA remain unclear. In this study, TRAIL receptor-Death receptor 4 (DR4), DR5, and LFA-1 ligand-intercellular adhesion molecule-1 (ICAM-1) were upregulated in IL-1β-stimulated HFLS-RA. We demonstrated that the total cell number of IL-1β-stimulated hUCMSCs adhering to IL-1β-stimulated HFLA-RA increased via LFA-1/ICAM-1 interaction. Direct co-culture of IL-1β-stimulated hUCMSCs with IL-1β-stimulated HFLS-RA increased the apoptosis of HFLS-RA. RA symptoms in the CIA mouse model improved after administration of IL-1β-stimulated hUCMSCs. In conclusion, IL-1β-stimulated hUCMSCs adhering to HFLS-RA occurred via LFA-1/ICAM-1 interaction, apoptosis of HFLS-RA was induced via TRAIL/DR4, DR5 contact, and RA symptoms and inflammation were significantly improved in a CIA mouse model. The results of this study suggest that IL-1β-stimulated hUCMSCs have therapeutic potential in RA treatment.

## Introduction

Rheumatoid arthritis (RA) is an autoimmune joint disease characterized by synovial proliferation and lymphocyte accumulation that leads to progressive damage of the periarticular and articular structure^1^. The synovium consists of fibroblast-like synoviocytes (FLSs) and macrophage-like synoviocytes (MLSs), with FLSs being the predominant cell type in the synovial intima. The hyperplasia of the synovium lining mainly consists of tissue-invading FLSs, infiltrating lymphocytes, and macrophages^2,3^. FLSs possess three aggressive features: tumor-like hyper-proliferation, apoptosis resistance, and high invasion ability^4^. The FLSs-intrinsic factors have been implicated in favoring FLSs proliferation or decrease apoptosis^1^. The expansion of RA FLSs might be due to increased proliferation and/or insufficient apoptosis. Thus, inducing apoptosis of RA FLSs could be a therapeutic approach.

Mesenchymal stem cells (MSCs) therapy for RA has been studied in recent years. The therapeutic potential of bone marrow-derived mesenchymal stem cells (BM-MSCs) has been investigated in the RA animal model. Intra-articular knee implantation of autologous BM-MSCs in phase I-II clinical trial reveals that it is safe and well tolerated^5^. Treatment of anti-rheumatic drugs plus human umbilical cord-derived mesenchymal stem cells (hUCMSCs) has been found to enhance the therapeutic efficacy for patients with active RA^6^. There is also some research that found that the proliferation and invasive behavior of synoviocytes could be suppressed by hUCMSCs via different cell signaling^7,8^. However, limited studies have shown that hUCMSCs possess therapeutic potential in the treatment of RA.

Mesenchymal stem cells (MSCs) exhibit anti-inflammatory and immunomodulatory effects, enhance tissue regeneration in injured sites, and migrate toward inflammation sites^9,10^. Preclinical and clinical studies have shown that MSCs possess therapeutic potential in immune-related diseases^11,12^. However, the poor viability and adhesion ability of MSCs need to be improved for clinical application^13^. It has been found that pre-treatment with cytokines or growth factors enhances MSCs migration and adhesion abilities^14^. In our previous study, we found that the migration ability of human umbilical cord-derived mesenchymal stem cells (hUCMSCs) was promoted by IL-1β via activating the MLCK pathway^15^. IL-1β induces MMP-1 expression to enhance hUCMSCs migration via PAR1 and G-protien-coupled signaling pathway^16^. IL-1β induces MMP-3 secretion via ERK1/2 pathway to enhance hUCMSCs migration^17^. Moreover, IL-1β induces LFA-1 protein expression through the p38 MAPK pathway in hUCMSCs, and improvs adhesion ability to endothelial cells via LFA-1/ICAM cell adhesive interaction^18^, as well as transendothelial migration ability^19^. We also found that IL-1β stimulated hUCMSCs could migrate to injured pancreas in diabetic mouse models^15^. These studies suggest that through the process of adhesion to endothelial cells and transendothelial migration, IL-1β stimulated hUCMSCs migrate to inflammation sites. The ability of hUCMSCs to migrate to inflammation sites may support the therapeutic potential of MSCs in cell therapy.

Lymphocyte function-associated antigen 1 (LFA-1, αLβ2), also known as CD11a/CD18, is a member of the integrin family^20^. LFA-1 is a key integrin that mediates cell-to-cell or cell-to-extracellular matrix adhesion and plays a critical role in leukocyte migration^21^. Adhesion molecules participate in rolling and adhering in the process of stem cells homing^22,23^. In our previous study, we found that the expression of LFA-1 is upregulated in IL-1β stimulated-hUCMSCs^18^. As the main ligand, Intercellular adhesion molecule 1 (ICAM-1) has high affinity to LFA-1^24^. ICAM-1 is widely expressed on the cell surface of different types of cells, such as leukocytes, endothelial cells, and epithelial cells^25^. The expression of ICAM-1 in FLSs can be enhanced by IL-1, tumor necrosis factor α (TNF-α), interferon-γ (IFN-γ), and IL-4 stimulation^26,27^. In FLS-RA, the expression of ICAM-1 is upregulated by IL-27 stimulation combining TNF-α or IL-1β^28^.

Tumor necrosis factor-related apoptosis-inducing ligand (TRAIL or Apo 2 ligand) belongs to the tumor necrosis factor (TNF) superfamily. In recent years, TRAIL has been applied in cancer therapy research because of its apoptosis-inducing potential. TRAIL induces cell apoptosis via binding with its receptors. TRAIL has five receptors, in which both death receptors TRAIL-R1 (DR4) and TRAIL-R2 (DR5) possess a death domain capable of inducing cell apoptosis^29–32^. By contrast, the other three receptors act as decoy receptors to inhibit apoptosis when overexpressed^31^. It has shown that RA FLSs express DR4 and DR5^33^. Previous studies have shown that TRAIL induces cell apoptosis in a variety of cancer cell lines, but exhibits no cytotoxicity to most normal human cells^29^. Moreover, TRAIL does not cause toxicity to tissues and organs by intravenous injection in nonhuman primates^29^. However, the short half-life of the recombinant form of TRAIL has limited the efficacy of its antitumor effects^34^. In our previous studies, TRAIL is upregulated after IL-1β stimulation in hUCMSCs and enhances apoptosis of breast cancer cells^35^. Thus, IL-1β stimulated hUCMSCs with enhanced TRAIL expression may be able to overcome the short half-life treatment barrier of TRAIL for therapeutic approaches.

In this study, to mimic the inflammatory microenvironment of RA joint tissue, the pro-inflammatory cytokine IL-1β was used to stimulate human fibroblast-like synoviocytes-rheumatoid arthritis (HFLS-RA). Afterward, the expression of ICAM-1, DR4, and DR5 in HFLS-RA were examined. In our previous study, we found that TRAIL expression in hUCMSCs can be enhanced by treatment with IL-1β. After IL-1β stimulation of HFLS-RA as well as of hUCMSCs, we further investigated whether the interaction between HFLS-RA and IL-1β stimulated hUCMSCs occurred via ICAM-1/LFA-1. The ability of IL-1β stimulated hUCMSCs to induce HFLS-RA cells apoptosis was also determined. In addition, the therapeutic efficacy of hUCMSCs stimulated with IL-1β in collagen-induced arthritis (CIA) mice was evaluated by histochemistry staining and by ^18^F-FDG microPET/MRI imaging.

## Results

### IL-1β stimulates ICAM-1 expression in HFLS-RA cells

**Figure 1:**
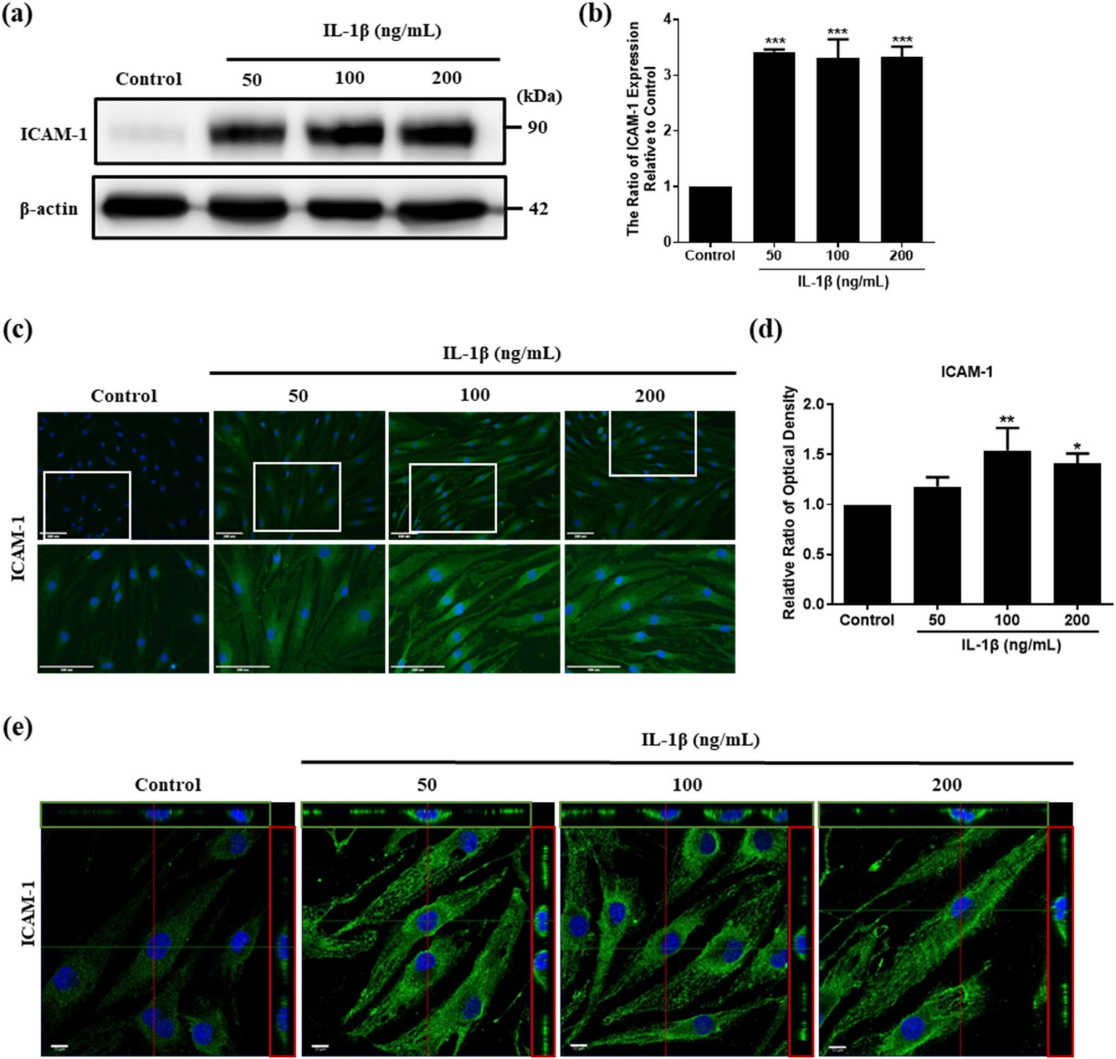
The effect of IL-1β stimulation on ICAM-1 expression in HFLS-RA cells. (**a**) HFLS-RA cells were stimulated with 50, 100, 200 ng/mL IL-1β for 24 h. The expression of ICAM-1 was examined by Western blot. (**b**) Quantitative results of the Western blot ICAM-1 expression of (**a**) (n = 3). (**c**) The images of immunofluorescence showed ICAM-1 expression in different concentrations of IL-1β stimulation for 24 h. White blocks chosen are magnified in the column below. Green: ICAM-1, blue: Hoechst 33258 (nucleus), scale bar: 100 μm. (**d**) Quantitative fluorescence intensity results of (**c**) analyzed by ImageJ. (**e**) Immunofluorescence images captured with Laser confocal microscope. Scale bar: 10 μm. The full-length of Western blot were in Supplementary Fig. S6. The data represent mean ± SD (*P < 0.05, **P < 0.01, ***P < 0.001).

ICAM-1 participates in many immunological response processes, including adhesion and transendothelial migration of immune cells to inflammation sites. As an adhesion molecule of HFLS-RA, we investigated ICAM-1 expression with IL-1β stimulation to mimic the inflammation environment in RA joints. After treatment with 50, 100, and 200 ng/mL IL-1β for 24 h, the results of Western blot showed that ICAM-1 was significantly increased on HFLS-RA (Fig. 1a,b). We also confirmed ICAM-1 expression on HFLS-RA after treatment with 100 ng/mL IL-1β for 6, 16, 24, 48 h by immunofluorescence. The results showed that ICAM-1 was upregulated with increasing IL-1β treatment time (Supplementary Fig. S1a,b). Interestingly, at the stimulation duration of 16 h, we found that most ICAM-1 expression was concentrated near the nucleus. At the stimulation duration of 24 h, ICAM-1 expression was more evenly distributed within the entire cell. It could be the newly synthesized ICAM-1 may translocate to the entire cell later. Using immunofluorescence staining, the results showed that ICAM-1 expressions were significantly increased after 100 ng/mL and 200 ng/mL IL-1β stimulation and that ICAM-1 had the highest expression at the concentration of 100 ng/mL IL-1β stimulation (Fig. 1c,d). Since ICAM-1 is a cell surface ligand of LFA-1, we examined the protein distribution on the cell surface by capturing images using Laser Confocal Microscope. The results showed that ICAM-1 expressions were increased on the cell surface after IL-1β stimulation (Fig. 1e).

### The role of IL-1β played in LFA-1/ICAM-1 interaction between hUCMSCs and HFLA-RA

**Figure 2:**
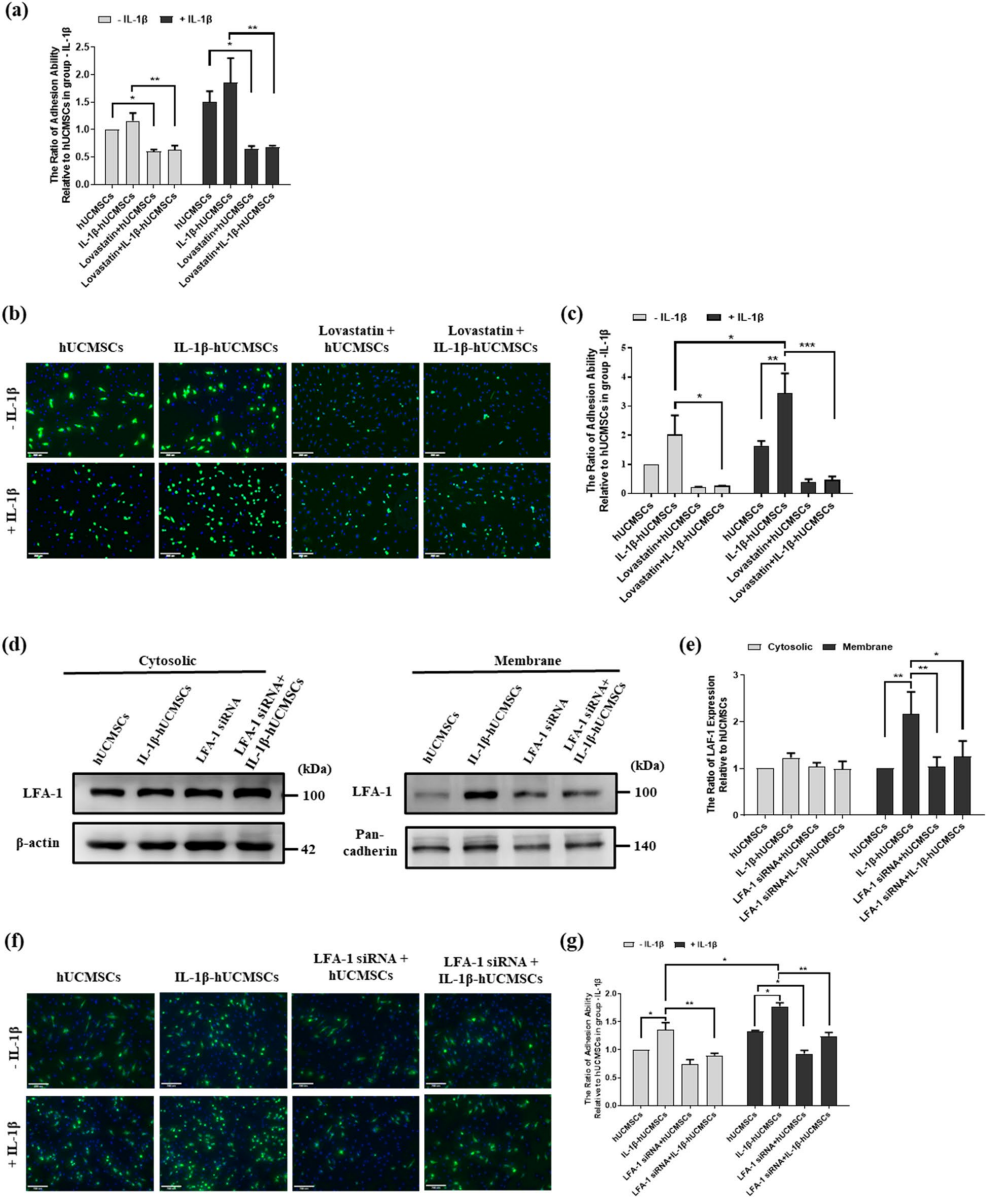
Effects of IL-1β in cell adhesion ability of hUCMSCs to HFLS-RA cells. (**a**) The adhesion assay of hUCMSCs to HFLS-RA cells with or without IL-1β stimulation (labeled with: +IL-1β and −IL-1β). HFLS-RA seeded in 96 wells, then Calcein AM labeled hUCMSCs were added in wells with/without Lovastatin for 1 h. The adhesion of hUCMSCs to HFLS-RA were detected by multimode microplate readers. (**b**) Using fluorescence study of cell adhesion assay for hUCMSCs and HFLS-RA cells with/without 100 ng/mL IL-1β stimulation, and with/without adding Lovastatin for 24 h then co-cultured for an hour. hUCMSCs were labeled with Calcein AM, and nucleus stained with Hoechst 33258. (**d**) hUCMSCs were with/without IL-1β stimulation and with/without LFA-1 siRNA transfection. The cytosolic and membrane protein were fractionated by Mem-PER™ Plus membrane protein extraction kit, then the expression of LFA-1 protein was detected by Western blot. β-actin was used as a cytosolic marker whereas pan-cadherin was used as a cell membrane marker. (**e**) Quantitative results of the Western blot ICAM-1 expression of (**d**). (**f**) hUCMSCs and HFLS-RA cells with/without 100 ng/mL IL-1β stimulation and with/without adding Lovastatin for 24 h, then co-cultured for an hour. hUCMSCs were labeled with Calcein AM, and nucleus stained with Hoechst 33258. (**c**,**g**) Quantitation of the cell number of five random fields (**b**,**f**) normalized with hUCMSCs without IL-1β stimulation, the results were analyzed by ImageJ. Scale bar: 100 μm. Full-length Western blots are in Supplementary Fig. S6. The data represent mean ± SD (n = 3) (*P < 0.05, **P < 0.01, ***P < 0.001).

LFA-1/ICAM-1 interaction plays an important role in leukocyte adhesion to endothelial cells in various inflammatory diseases. Here, proinflammatory cytokine IL-1β was used to mimic the inflammation microenvironment. We investigated the interaction between hUCMSCs and HFLA-RA after IL-1β stimulation by cell adhesion assay. At first, we examined the cell viability in both hUCMSCs and HFLA-RA cells treated with IL-1β and LFA-1 inhibitor-Lovastatin. The MTT assay showed that the cell viability was not affected in hUCMSCs after treatment with IL-1β and different concentrations of Lovastatin (Supplementary Fig. S2a). However, cell viability of HFLS-RA cells was significantly decreased by treatment with 100 μM Lovastatin (Supplementary Fig. S2b). Based on MTT assay, we used 50 μM Lovastatin as our final concentration. Then, we determined the role that IL-1β plays in LFA-1/ICAM-1 interaction between hUCMSCs and HFLS-RA cells. Adhesion assay showed that there were only a small number of hUCMSCs adhered in the group without IL-1β stimulated HFLS-RA cells. On the contrary, IL-1β stimulation significantly increased the number of adhering hUCMSCs to HFLS-RA. In both groups of HFLS-RA with or without IL-1β stimulation, the number of adhering hUCMSCs was significantly decreased in the presence of Lovastatin (Fig. 2a). This indicates that blocking LFA-1 in HFLS-RA reduces hUCMSCs adhesion to HFLS-RA.

We also examined the LFA-1/ICAM-1 interaction by using immunofluorescence study. In the group of HFLA-RA without IL-1β stimulation, the number of IL-1β stimulated hUCMSCs adhered to HFLS-RA was higher than hUCMSCs. In the group of HFLS-RA with IL-1β stimulation, adhesion to both hUCMSCs and IL-1β stimulated hUCMSCs significantly increased. Consistent with the adhesion assay results, the number of adhering hUCMSCs was significantly decreased in the presence of Lovastatin in both groups of HFLS-RA cells with or without IL-1β stimulation (Fig. 2b,c). We further examined the effect of IL-1β on LFA-1 expression in hUCMSCs by Western blot. The data showed that IL-1β upregulates LFA-1 expression in hUCMSCs cell membrane. siRNA-LFA-1 transfection of hUCMSCs resulted in knockdown of IL-1β induced LFA-1 protein levels (Fig. 2d,e). With or without IL-1β stimulation, the number of hUCMSCs adhering to HFLS-RA was significantly increased and reduction of LFA-1 expression by siRNA-LFA-1 suppressed IL-1β-induced cell adhesion of hUCMSCs with HFLA-RA (Fig. 2f,g). These results suggested that IL-1β plays a vital role in promoting hUCMSCs adhesion to HFLA-RA cells through LFA-1/ICAM-1 interaction.

### IL-1β stimulates DR4 and DR5 expression in HFLS-RA cells

**Figure 3:**
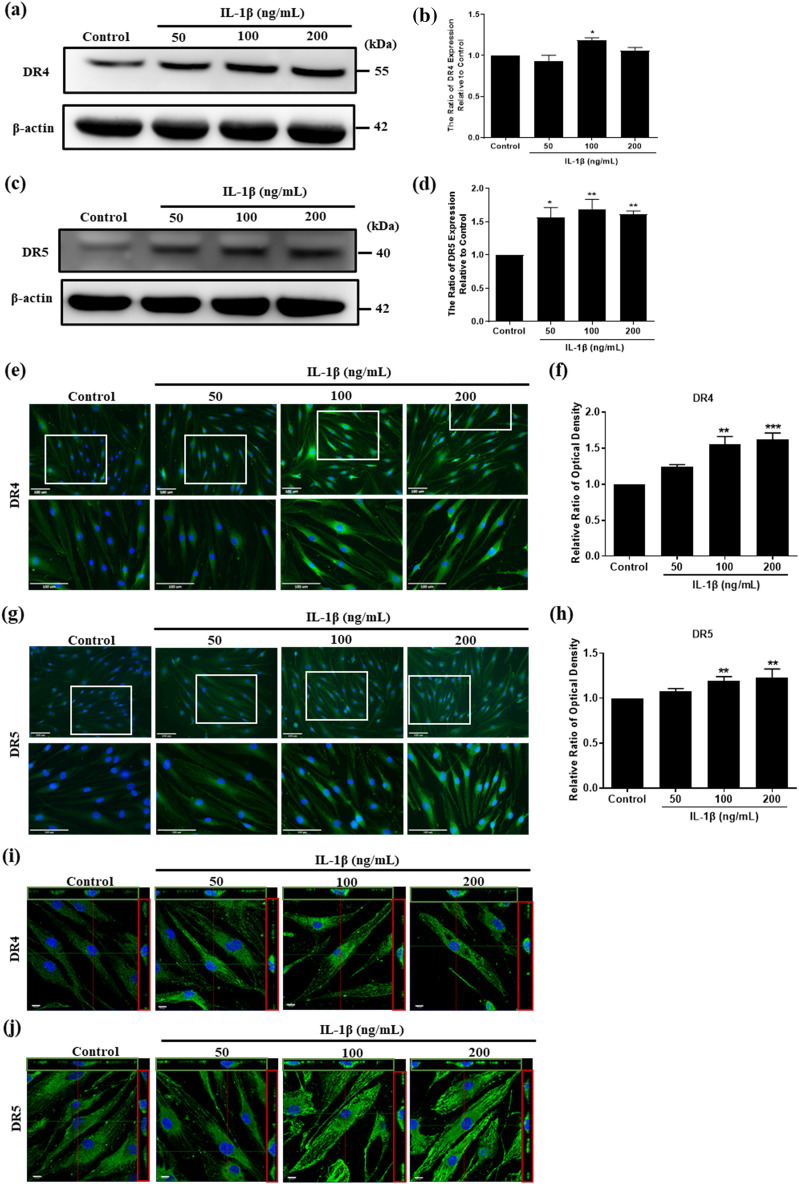
The effect of IL-1β stimulation on DR4 and DR5 expression in HFLS-RA cells. HFLS-RA cells were stimulated with 50, 100, 200 ng/mL IL-1β for 24 h. The expression of (**a**) DR4 and (**c**) DR5 were examined by Western blot. (**b**) Quantitative results of the Western blot DR4 expression of (**a**). (**d**) Quantitative results of the Western blot DR5 expression of (**c**). The expression of (**e**) DR4 and (**g**) DR5 were examined by immunofluorescence. White blocks chosen are magnified in the column below. Green: DR4 or DR5, blue: Hoechst 33258 (nucleus), scale bar: 100 μm. (**f**,**h**) Quantitative fluorescence intensity results of (**e**,**g**) analyzed by ImageJ. Immunofluorescence images of (**i**) DR4 and (**j**) DR5 expression captured by using Laser confocal microscope. Scale bar: 10 μm. Full-length Western blots are in Supplementary Fig. S6. The data represent mean ± SD (n = 3) (*P < 0.05, **P < 0.01, ***P < 0.001).

HFLS-RA are reported to express TRAIL receptors DR4 and DR5. We investigated the DR4 and DR5 expression in HFLS-RA treated with 100 ng/mL IL-1β for 6, 16, 24, 48 h by immunofluorescence. The results showed that the protein levels of DR4 and DR5 were upregulated with increased IL-1β treatment time. The protein level of DR4 exhibited the maximum expression at 24 h of IL-1β treatment (Supplementary Fig. S3a,b). As for DR5, the protein level was induced after IL-1β treatment for 24 h (Supplementary Fig. S3c,d). Thus, to further identify the effect of IL-1β on DR4 and DR5 expression, HFLS-RA cells were stimulated with 50, 100 and 200 ng/mL IL-1β for 24 h. The results of Western blot showed that both DR4 and DR5 were upregulated after IL-1β stimulation. At the concentration of 100 ng/mL IL-1β, both DR4 and DR5 expressions were upregulated (Fig. 3a–d). We also confirmed IL-1β induced DR4 and DR5 expression by immunofluorescence. The results showed that DR4 and DR5 were significantly upregulated after 100 and 200 ng/mL IL-1β stimulation (Fig. 3e–h). Since DR4 and DR5 are receptors, we examined the functional protein whether expressed on the cell surface or not by capturing the images using Laser Confocal Microscope. The results showed that DR4 and DR5 expression were increased on the cell surface after IL-1β stimulation (Fig. 3i,j).

### TRAIL expression induced by IL-1β in hUCMSCs cells

**Figure 4:**
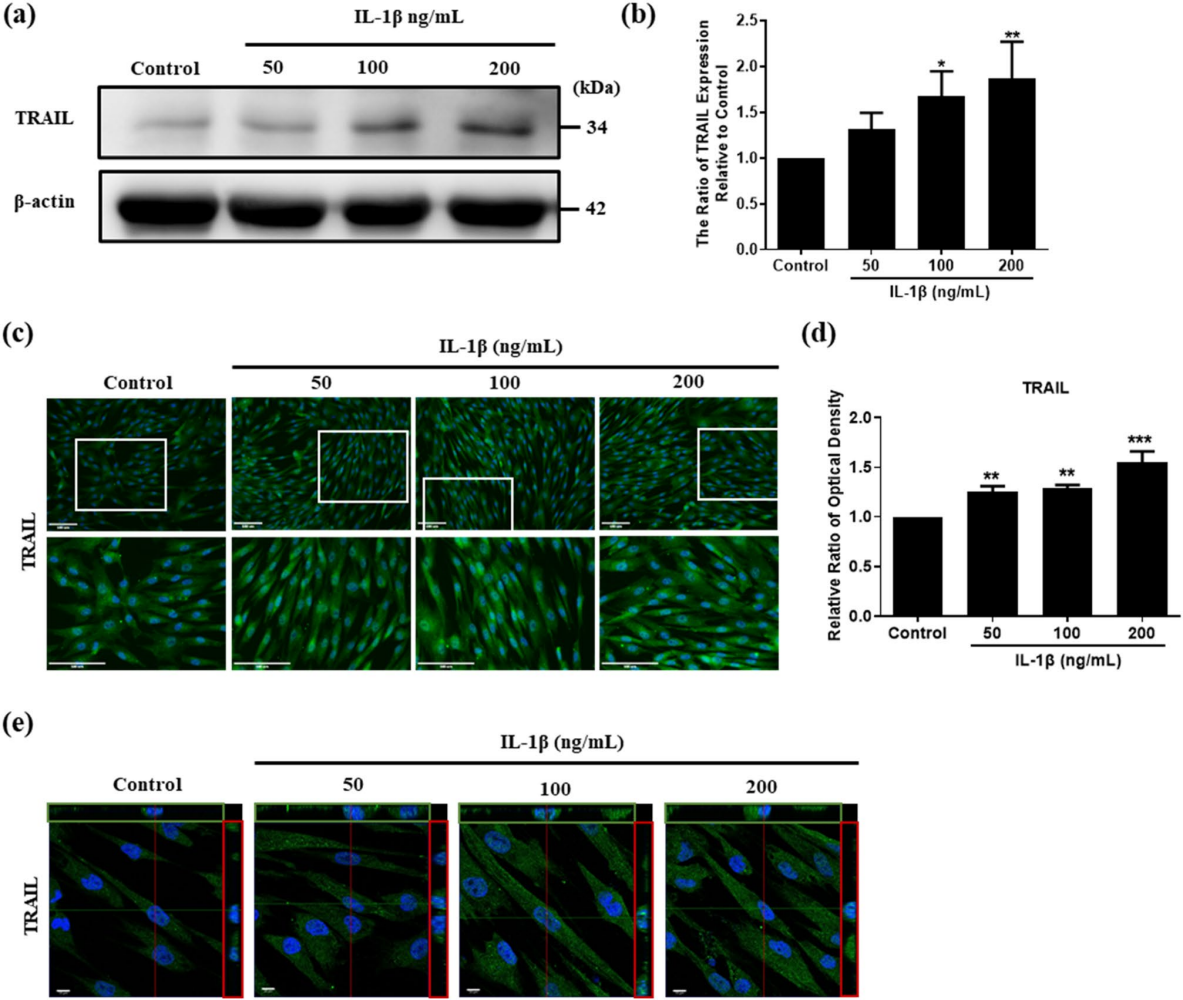
The expression of TRAIL with different IL-1β concentration stimulation in hUCMSCs. (**a**) hUCMSCs were stimulated with 50, 100, 200 ng/mL IL-1β for 24 h. The expression of TRAIL was examined by Western blot. (**b**) Quantitative results of the Western blot TRAIL expression of (**a**). (**c**) The images of immunofluorescence showed TRAIL expression in different concentrations of IL-1β stimulation for 24 h. White blocks chosen are magnified in the column below. Green: TRAIL, blue: Hoechst 33258 (nucleus), scale bar: 100 μm. (**d**) Quantitative fluorescence intensity results of (**c**) analyzed by ImageJ. (**e**) Immunofluorescence images of TRAIL expression captured using Laser confocal microscope. Scale bar: 10 μm. Full-length Western blots are in Supplementary Fig. S6. The data represent mean ± SD (n = 3) (*P < 0.05, **P < 0.01, ***P < 0.001).

To evaluate the effect of IL-1β in TRAIL expression, hUCMSCs were stimulated with 50, 100, and 200 ng/mL IL-1β for 24 h. The Western blot results showed that 100 and 200 ng/mL of IL-1β caused an upregulation of TRAIL expression (Fig. 4a,b). We further confirmed TRAIL expression after IL-1β stimulation on hUCMSCs using immunofluorescence. The results showed that TRAIL was upregulated after IL-1β stimulation in hUCMSCs (Fig. 4c,d). As TRAIL is a member of type II transmembrane protein, we evaluated the functional protein expressed on cell surface using Laser Confocal Microscope to capture the images. The results showed that TRAIL increased on the cell surface after IL-1β stimulation (Fig. 4e).

### Induce HFLS-RA apoptosis after direct co-cultured with hUCMSCs

**Figure 5:**
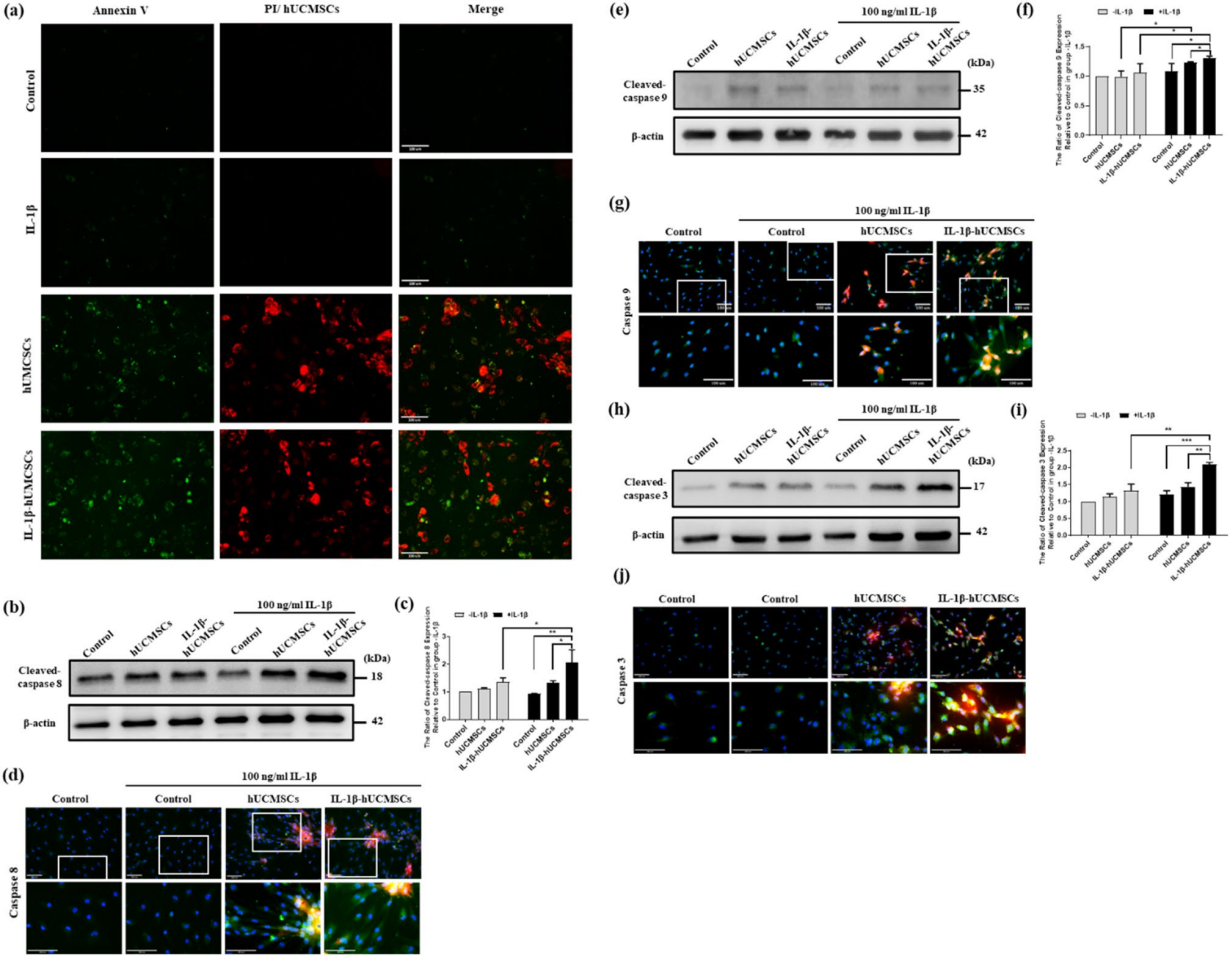
The effects of IL-1β stimulated hUCMSCs induced in HFLS-RA cells apoptosis and caspases activation. (**a**) After 24 h of co-culture, cells were stained with Annexin V/PI detection kit. hUCSMCs were labeled with CellTracker Orange. Annexin V represented as early apoptosis, and PI represented as late apoptosis. Green: Annexin V, red: nucleus-PI, whole cell with light red—hUCMSCs, scale bar: 100 μm. (**b**) Cleaved caspase-8, (**e**) cleaved caspase-9, and (**h**) cleaved caspase-3 expression in HFLS-RA cells and HFLS-RA cells co-cultured with hUCMSCs. The quantitative graphs of the Western blot results of (**c**,**f**,**i**). Full-length Western blots are in Supplementary Fig. S6. The data represent mean ± SD (n = 3) (*P < 0.05, **P < 0.01, ***P < 0.001). The expression of (**d**) cleaved caspase-8, (**g**) cleaved caspase-9, and (**j**) cleaved caspase-3 expression on HFLS-RA cells after co-culturing with hUCMSCs by immunofluorescence study. White blocks chosen are magnified in the column below. Orange: hUCMSCs labeled with CellTracker Orange, Green: cleaved caspase, blue: Hoechst 33258 (nucleus), scale bar: 100 μm.

As TRAIL is involved to induce TRAIL-related apoptosis by binding to DR4 and DR5, HFLS-RA apoptosis induced by co-culture with hUCMSCs was examined using Annexin V/PI detection kit. Due to the similarity of color used in labeling HFLS-RA cells and Celltracker orange labeled hUCMSCs, only annexin V-positive cells can be confirmed as the apoptotic HFLS-RA cells. The results showed that apoptosis was not increased with IL-1β stimulation in HFLS-RA cells. However, apoptotic HFLS-RA cells did increase after coculture with hUCMSCs or with IL-1β stimulated hUCMSCs (Fig. 5a).

### Effects of IL-1β stimulated hUCMSCs on caspases expression of HFLS-RA cells

Research indicates that there are two main apoptotic pathways, the extrinsic pathway and the intrinsic pathway^36^. As TRAIL could induce the signaling pathway of TRAIL-related apoptosis, both hUCMSCs and HFLS-RA with or without IL-1β stimulation were directly co-cultured for 24 h to determine which apoptotic pathway is involved in hUCMSCs induced apoptosis of HFLS-RA. Then Western blot was used to examine the expression of cleaved-caspase-8, cleaved-caspase-9 and cleaved-caspase-3. The results showed that the protein expression of cleaved-caspase-8, cleaved-caspase-9 and cleaved-caspase-3 are significantly higher when IL-1β stimulated hUCMSCs were cocultured with IL-1β stimulated HFLS-RA cells in comparison to HFLS-RA cells co-cultured with hUCMSCs (Fig. 5b,c,e,f,h,i). The effects of the IL-1β stimulated hUCMSCs on caspase expression of HFLS-RA cells were also examined using immunofluorescence staining. The results showed that the expression of caspase-8, -9 and-3 in HFLS-RA increased after co-culture with hUCMSCs. These caspase expressions were more obvious in HFLS-RA after co-culture with hUCMSCs and IL-1β stimulated hUCMSCs (Fig. 5d,g,j). At 40 × magnification, we observed that HFLS-RA cells around hUCMSCs and IL-1β stimulated hUCMSCs increased the expression of caspase-8, -9 and -3. The expression of caspase-8, -9 and -3 are highly increased in HFLS-RA cells around IL-1β stimulated hUCMSCs (Fig. 5c,f,i). The results suggest that HFLS-RA cells apoptosis were induced by hUCMSCs via cell–cell contact. Both extrinsic and intrinsic apoptotic pathways are involved in IL-1β stimulated hUCMSCs induced HFLS-RA cells apoptosis.

### Therapeutic efficacy evaluation of hUCMSCs administration in CIA mouse model

**Figure 6:**
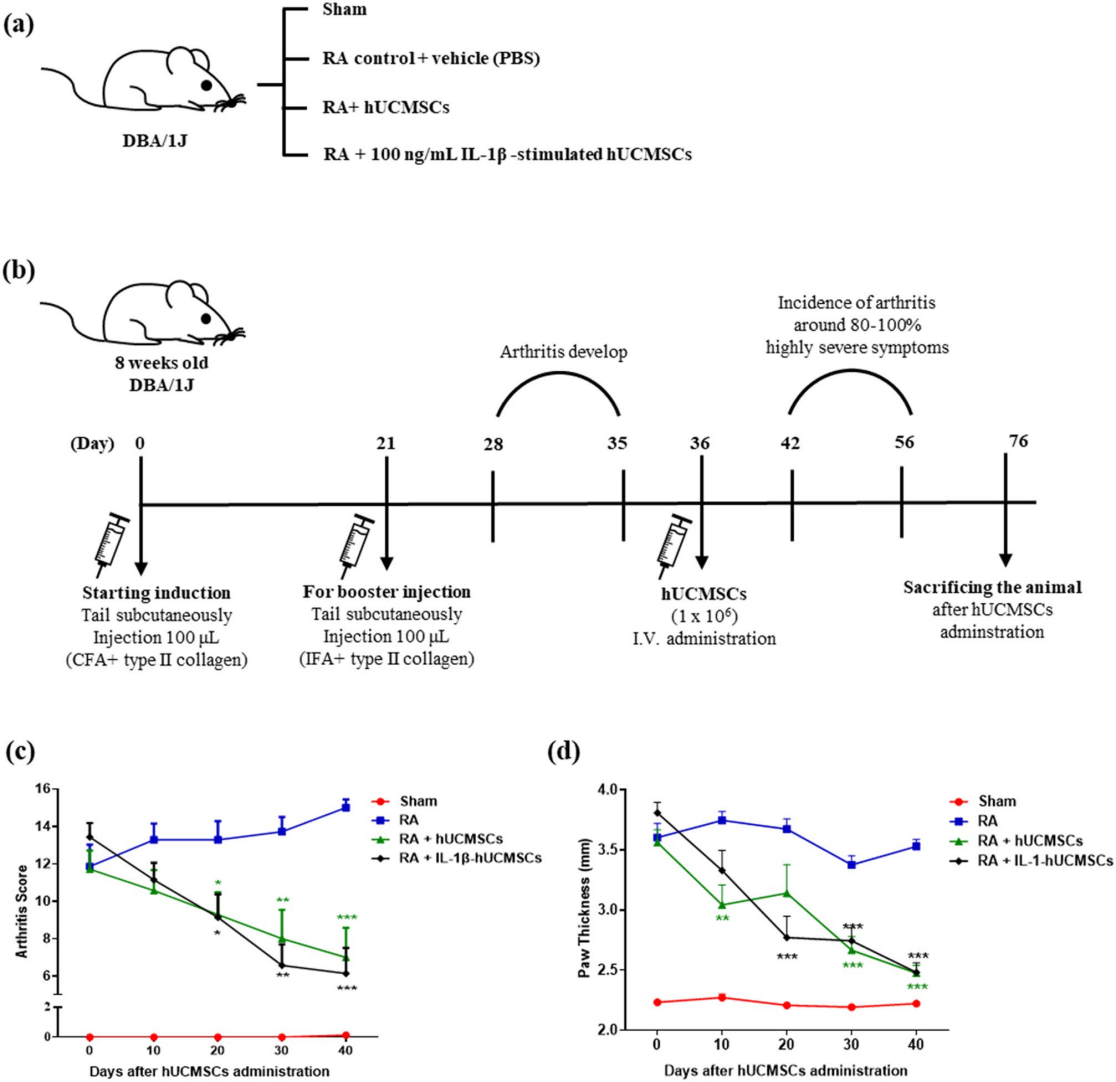
Therapeutic efficacy evaluation of hUCMSCs in CIA mouse model. (**a**) The group of CIA mouse models. Sham: without collagen induction, RA: CIA mice without hUCMSCs administration, hUCMSCs: CIA mice with 1 × 10^6^ hUCMSCs administration, IL-1β stimulated hUCMSCs: CIA mice with 1 × 10^6^ IL-1β stimulated hUCMSCs. (**b**) The timeline of RA induction and hUCMSCs administration. Mice were randomly divided into four groups on day 36 after the first immunization. (**c**) Statistical results of arthritis score after hUCMSCs administration. (**d**) Statistical results of paw thickness after hUCMSCs administration. The data represent mean ± SD (n = 7 per group) (*P < 0.05, **P < 0.01, ***P < 0.001).

To evaluate the therapeutic efficacy of hUCMSCs, we recorded the data of body weight, arthritis score, and paw thickness. The grouping and the timeline of CIA induction and hUCMSCs administration are represented as in Fig. 6a,b. There was no significant change in body weight from day 0 to day 40 after hUCMSCs administration (Supplementary Fig. S4a). The results show that the arthritic score of the RA group was gradually increased. Compared to the RA group, the arthritis score of both hUCMSCs and IL-1β stimulated hUCMSCs administration groups were significantly decreased (Fig. 6c). As for the evaluation of paw thickness, the thickness of hUCMSCs and IL-1β stimulated hUCMSCs administration groups significantly decreased in comparison to the RA group (Fig. 6d). These results indicate that the paw swelling of CIA mice significantly improved after hUCMSCs and IL-1β stimulated hUCMSCs administration. The therapeutic efficacy was also examined by photographing the exterior appearance of the front and hind paws. We found that mice in the RA group exhibited more redness and severe swelling in comparison to the sham group. However, RA symptoms were improved with hUCMSCs and IL-1β stimulated hUCMSCs administration on day 40 in comparison to the RA group (Supplementary Fig. S4b).

### Evaluated the effect of hUCMSCs in inflammation of CIA mice with ^18^F-FDG microPET/CT imaging

**Figure 7:**
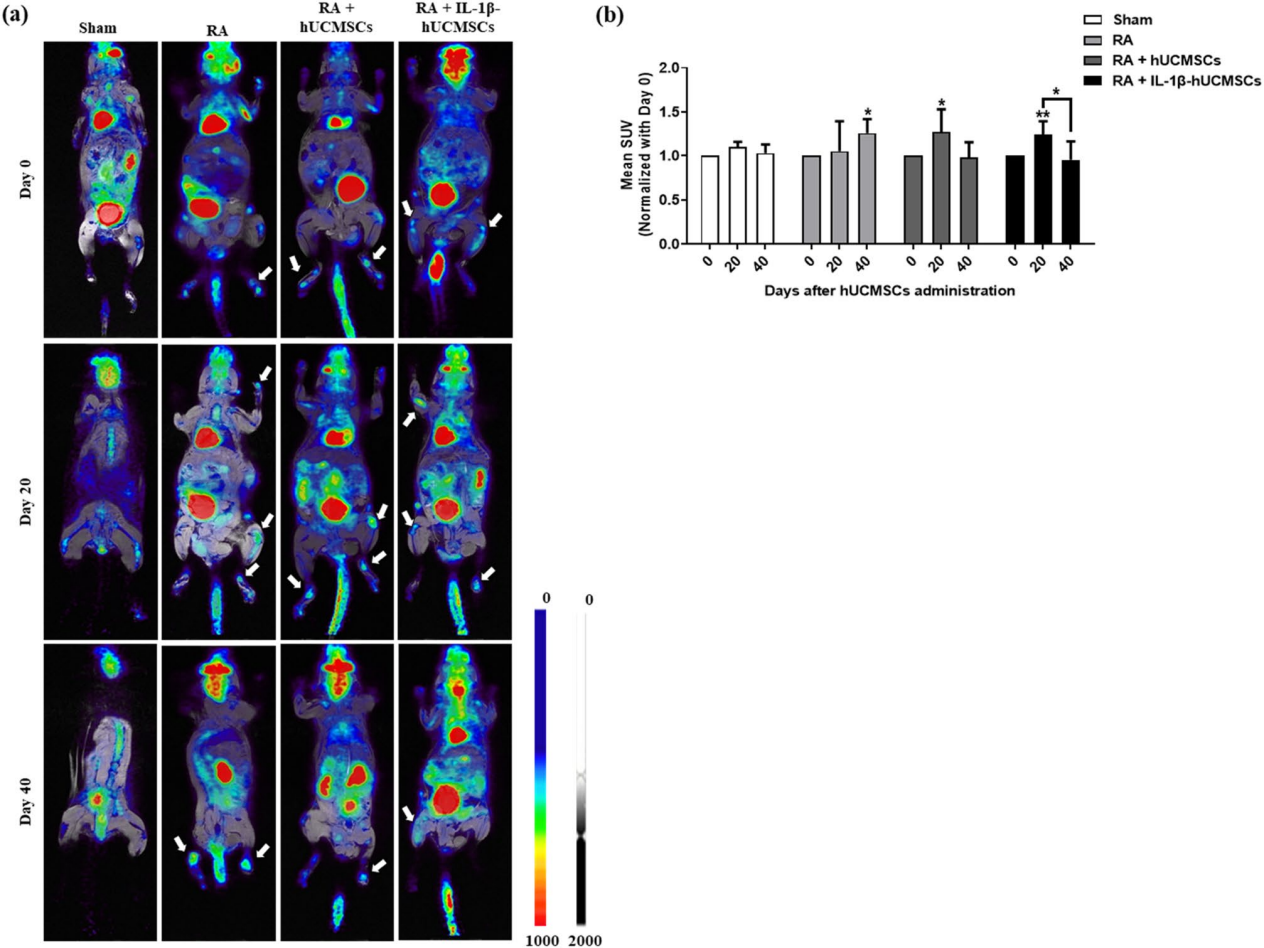
Effect of hUCMSCs administration to the uptake of ^18^F FDG in the limbs of CIA mice. (**a**) On day 0, 20 and 40, ^18^F-FDG microPET/CT was used for monitoring severity of inflammatory disease activity in RA mice. The uptake of ^18^F-FDG in the limbs of sham, RA and RA after administering hUCMSCs or IL-1β stimulated hUCMSCs mice. Both administration of hUCMSCs and IL-1β stimulated hUCMSCs suppress the uptake of ^18^F-FDG in the ankles and metatarsal joints of RA-bearing mice. Arrow: Severely inflamed locations in the limbs. (**b**) Compared with Day 0 in each group, the accumulated ^18^F-FDG show that the standardized uptake value (SUV) decreased after administering hUCMSCs or IL-1β stimulated hUCMSCs for 40 days. The data represent mean ± SD (n = 5 per group) (*P < 0.05, **P < 0.01).

^18^F-FDG has been used to detect inflammatory disorders. Here ^18^F-FDG microPET/CT was performed to monitor the inflammatory activity in CIA mice joints. After administering hUCMSCs or IL-1β stimulated hUCMSCs to CIA mice for 40 days, the ^18^F-FDG uptake in the limbs of CIA mice were suppressed (Fig. 7a). Using Amide’s Medical Imaging Data Examiner (AMIDE) software, the regions of interest (ROI) were selected and the accumulated ^18^F-FDG measured. The results show that the standardized uptake value (SUV) decreased after administering hUCMSCs or IL-1β stimulated hUCMSCs for 40 days (Fig. 7b). These data suggested both hUCMSCs and IL-1β stimulated hUCMSCs are capable of suppressing inflammation in CIA mice joints.

### Evaluation of bone erosion and synovial hyperplasia in CIA mouse model

**Figure 8:**
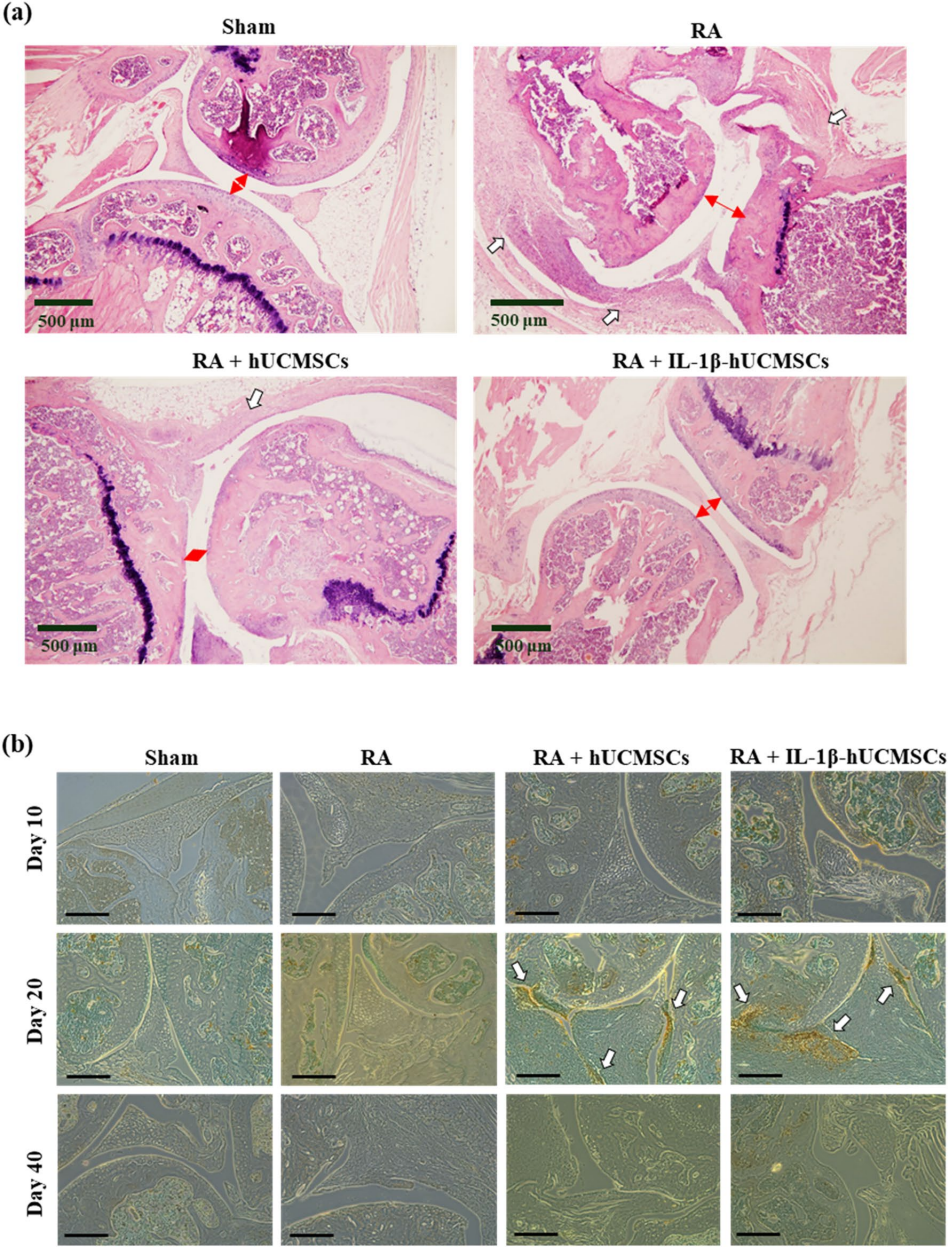
The therapeutic efficacy of hUCMSCs was evaluated by H&E stain and TUNEL assay in CIA Mouse Model. (**a**) CIA mice were sacrificed at day 40 after hUCMSCs administration. The joint tissues were collected and the pathology of synovial hyperplasia and bone destruction examined. Scale bar: 500 μm. White arrow head: synovial hyperplasia. Red arrow head: the distance of the joint cavity. (**b**) The apoptotic cells in the synovial joint tissue sections after administration of hUCMSCs and IL-1β stimulated hUCMSCs were examined by using TUNEL Assay Kit. Arrow: the staining of apoptotic cells region. Scale bar: 250 μm.

In order to confirm the therapeutic efficacy of hUCMSCs administration, joint tissues were examined for pathological analysis by H&E stain. Mice were sacrificed on day 40 after hUCMSCs administration, the degree of bone erosion and synovial hyperplasia were examined. The results showed that in comparison to the sham group, less cartilage was preserved, and severe synovial hyperplasia was found in the RA group. The results also show that the synovium invaded the bone area and damaged the synovial joint (Fig. 8a). With administration of hUCMSCs and IL-1β stimulated hUCMSCs, more cartilage was preserved and synovial hyperplasia was ameliorated (Fig. 8a). Furthermore, the cell apoptosis in the synovial joint after administration of hUCMSCs and IL-1β stimulated hUCMSCs were examined by TUNEL Assay Kit. The results showed that in comparison with the control and RA groups, more cell apoptosis was observed in joint tissue sections of hUCMSCs and IL-1β stimulated hUCMSCs administered for 20 days (Fig. 8b). We also observed the apoptotic fibroblast-like synoviocytes in hyperplasia region (Supplementary Fig. S5). Combined with the result of microPET/CT imaging, H&E stain, and TUNEL assay, we could infer that the occurrence of apoptosis reduces the severity of arthritis.

## Discussion

HFLSs play a critical role in RA due to its unique aggressive behavior damaging the joints^37^. Migration and invasion of synoviocytes seem to be the main cause of bone erosion. It has been found that FLSs-RA have CC and CXC chemokine receptors to mediate cells’ proliferation, migration, and MMP production^38^. Moreover, the hyperplasia of synovium is also a critical issue in RA because FLSs have the ability of apoptosis resistance^37^. Therefore, the regulation of the proliferation and apoptosis of FLSs should effectively prevent the articular destruction in RA. A recent study shows that FLSs express TRAIL receptors DR4 and DR5, which have a death domain capable of inducing cell apoptosis^39^. In our previous study, we found that with IL-1β stimulation, the expression of TRAIL in hUCMSCs was increased^35^. Thus, we speculated that TRAIL induced by IL-1β stimulated hUCMSCs might be able to induce the apoptosis of FLSs-RA to ameliorate RA symptoms. In this study, the adhesion of IL-1β stimulated hUCMSCs to FLSs via LFA-1/ICAM-1 interaction, and the IL-1β stimulated hUCMSCs induced FLS apoptosis via TRAIL/DR4, DR5 contact were determined. The therapeutic efficacy of IL-1β stimulated hUCMSCs administration to the CIA mouse model was also evaluated.

The therapeutic efficacy of MSCs have been reported in RA treatment^7,40^. However, research seldom focuses on the potential of hUCMSCs^41^ and rarely discusses the mechanism of hUCMSCs homing to inflammation joints and interaction with FLSs. As a pro-inflammatory cytokine, IL-1β plays a vital role in inducing migration, proliferation, differentiation, and apoptosis in different cells^15,42–44^. Previous studies have shown that IL-1β induces human BM-MSCs migration and leucocyte chemotaxis^45^. Various cytokines are involved in RA inflammatory response, IL-1β, one of the members who participate in synovial proliferation and joint destruction, is produced by macrophage and dendritic cells^46,47^. IL-1β has been applied to mimic the pro-inflammatory environment of FLSs proliferation and acts as a mediator to stimulate MMPs secretion^48,49^.

It has been found that MSCs have homing ability to inflammation sites^15,45^. We found that the transendothelial migration abilities for homing to target sites was enhanced in hUCMSCs after stimulation with IL-1β^19^. Thus, the application of the ability of hUCMSCs to migrate to sites of inflammation could be a potential therapeutic to treat RA. In our previous study, we found that different concentrations of IL-1β significantly increased the expression of LFA-1 in hUCMSCs^18^. By treating HFLS-RA with IL-1β to mimic the inflammatory microenvironment in RA joints, the LFA-1 ligand ICAM-1 was highly expressed on the cell surface of HFLS-RA (Fig. 1). In an adhesion assay, we found that IL-1β stimulation increased the number of adhering cells. However, the number of adhering cells significantly decreased with the presence of LFA-1 antagonist-Lovastatin or siLFA-1 (Fig. 2). The results indicated that the adhesion mechanism of HFLS-RA cells with hUCMSCs occurs via ICAM-1/LFA-1 interaction.

TRAIL receptors DR4 and DR5 expression have been found in FLSc^39^. To understand the effect of the inflammatory environment on HFLS-RA and DR4 and DR5 expression, IL-1β was added to cultured HFLS-RA cells. We found that the expression of DR4 and DR5 was upregulated after IL-1β stimulation by Western blot and immunofluorescence study (Fig. 3). As for the ligand—TRAIL, the expression was also increased with IL-1β stimulation in hUCMSCs (Fig. 4). In order to evaluate the apoptosis-inducing ability of TRAIL-expressing hUMCSCs, cells were co-cultured with HFLS-RA cells detected by Annexin V/PI detection. The results showed that with the presence of hUCMSCs, Annexin V was stained significantly, and PI was also stained in the nucleus in HFLS-RA cells (Fig. 5). These results indicate that hUCMSCs induced early apoptosis and late apoptosis in HFLS-RA cells.

TRAIL ligand activates cell apoptosis pathways via contact with death receptors through the extrinsic pathway or the intrinsic pathway^50,51^. It has been found that TRAIL inducing RA-FLSs apoptosis is mediated by caspase-8^52^. Extrinsic pathway activates caspase-8, and finally activates caspase-3 causing cell apoptosis. In this study, co-culturing hUCMSCs with HFLS-RA cells induced the apoptosis of HFLS-RA cells (Fig. 5). The cleaved-caspase-8, -9 and -3 expressions were upregulated in HFLS-RA cells. By immunofluorescence staining, cleaved-caspase-8, -9 and -3 were found to be highly expressed in HFLS-RA cells which were around IL-1β stimulated hUCMSCs (Fig. 5). This indicates that hUCMSCs induced HFLS-RA apoptosis via cell–cell contact.

In a clinical trial, BM-MSCs were shown to be safe and well tolerated in intra-articular knee implantation^5^. In our study, hUCMSCs were administered via intravenous injection in the CIA mouse model to evaluate therapeutic efficacy. Human umbilical cord blood MSCs (hUCB-MSCs) have been found to alleviate RA symptoms by mediating macrophage polarization and inhibiting the activation of inflammasome^53^. Moreover, hUCMSCs improve the immune-associated prothrombotic state and promote articular recovery in the CIA rat model^54^. Recently, we found that macrophages co-cultured with IL-1β stimulated hUCMSCs could enhance M2 macrophage polarization and M2 macrophage apoptosis. In the CIA mice model, the imbalance of M1/M2 ratio in joints could be rehabilitated after injection of IL-1β-stimulated hUCMSCs^55^. This finding is consistent with the result in this study that both hUCMSCs and IL-1β stimulated hUCMSCs are capable of suppressing inflammation in CIA mice joints (Fig. 7). With hUCMSCs treatment, the symptoms of redness and swelling in their front and hind paws were significantly reduced and more cartilage was preserved (Fig. 8a). Furthermore, after administration of hUCMSCs and IL-1β stimulated hUCMSCs to CIA mice for 20 days, cell apoptosis can be found in the synovial joints (Fig. 8b). The fibroblast-like apoptotic cells can be observed at higher magnification of the apoptosis region in the hyperplasia of the RA + hUCMSCs and RA + IL-1β-hUCMSCs groups (Supplementary Fig. S5). To detect the administered hUCMSCs or IL-1β-hUCMSCs in these tissue sections, anti-human CD105 antibody and anti-human nucleus antibody were used for immunostaining. We did not found hUCMSCs in these sections. The intravenous delivery of MSCs have been examined by different cell marking techniques to trace the biodistribution of MSCs within 5 days^56,57^. For longer term tracing, the signal of intravenous injected luciferase and green fluorescent protein label MSCs were not detected after 14 days in histology analysis^58^. In our tissue sections, no intravenous tail vein injected hUCMSCs after 20 days were detected. These could be due to the limited number of hUCMSCs we injected (1 × 10^6^ cells), or could be due to the fact that transplanted cells can survive less than 20 days and gradually disappear in CIA mice. Based on the finding of our in vitro data and the in vivo data in Fig. 8 and Supplementary Fig. S5, we speculate that the intravenous tail vein administered IL-1β stimulated hUCMSCs migrate to the inflammation region of the joint to adhere to HFLSs and then secrete TRAIL to induce apoptosis of HFLSs in the CIA mouse model. However, tracking the long term activity of injected cells and co-localization for the intended purpose of relieving CIA symptoms should be explored in the further research.

**Figure 9:**
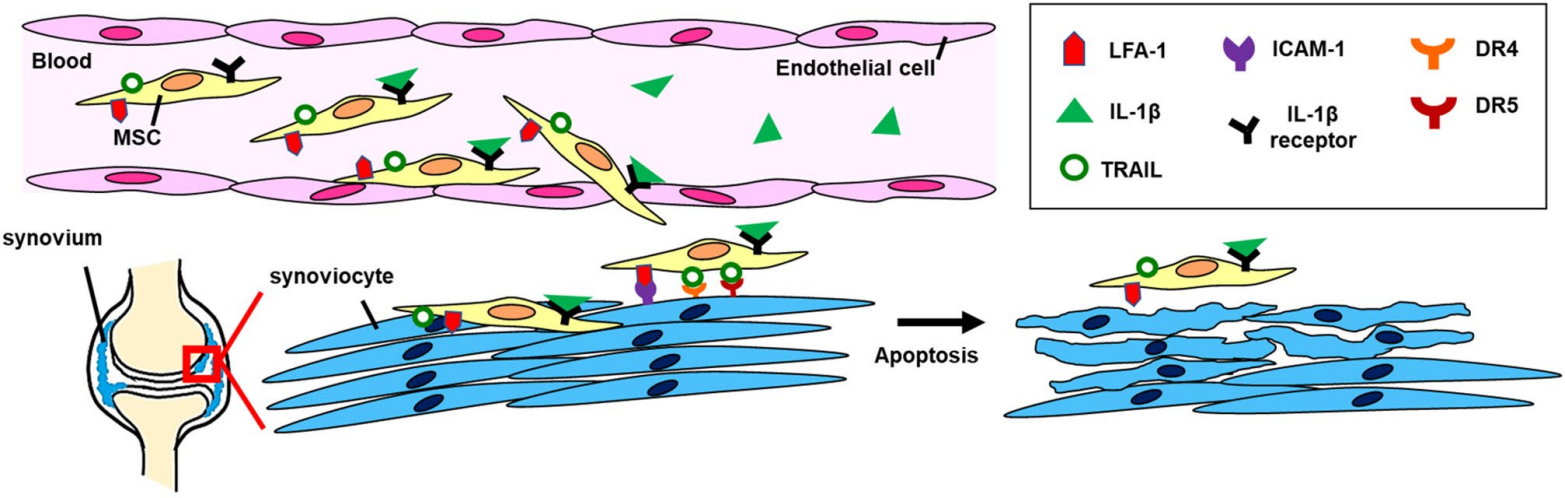
Schematic diagram of hUCMSCs inducing FLSs apoptosis in RA. A schematic diagram depicted the role of hUCMSCs homing to inflammation sites and interacting with FLSs via LFA-1/ICAM-1, and inducing FLSs apoptosis in RA.

In summary, our data suggest that the expression of DR4, DR5, and ICAM-1 were upregulated with IL-1β stimulation in HFLS-RA cells. TRAIL expression in hUCMSCs was enhanced after IL-1β stimulation. Via LFA-1/ICAM-1 interaction, IL-1β stimulated hUCMSCs adhere to HFLS-RA cells, and TRAIL induces HFLS-RA cells apoptosis (Fig. 9). In the CIA mouse model, RA symptoms and inflammation were significantly improved with IL-1β stimulated hUCMSCs administration. As a result, we suggest that IL-1β stimulated hUCMSCs possess several advantages for use in RA treatment. It is worth investigating for applications in clinical trials.

## Methods

### Human umbilical cord mesenchymal stem cells (hUCMSCs)

hUCMSCs were obtained from Bioresource Collection and Research Center (BCRC), Hsinchu, Taiwan. hUCMSCs were maintained in low serum defined medium, which consisted of 56% low-glucose Dulbecco’s Modified Eagle Medium (DMEM-LG; Invitrogen, CA, USA), 37% MCBD 201 (Sigma, MO, USA), 2% fetal bovine serum (Thermo, Logan, UT), 0.5 mg/mL of AlbuMAX^®^ I (Life technology, NY, USA), 1X Insulin-Transferrin-Selenium-A (Life technology, NY, USA), 10 nM Dexamethasone (Sigma, MO, USA), 10 ng/mL Epidermal growth factor (PeproTech, NJ, USA), 50 nM L-ascorbic acid 2-phosphate (Sigma, MO, USA), and 1 ng/mL of platelet-derived growth factor-BB (PeproTech, NJ, USA). The cells were incubated in a humidified chamber with 5% CO_2_ at 37 °C. Detaching with HyClone^®^ HyQtase (GE, UT, USA) and replated at a ratio 1:4 when cells reached 70–80% confluence.

### Human fibroblast-like synoviocytes: rheumatoid arthritis (HFLS-RA)

HFLS-RA were purchased from Cell Applications, INC., (San Diego, CA, USA), and cultured in Synoviocyte Growth Medium (Cell Applications, CA, USA) according to the general instructions for culturing. The cells were incubated in a humidified chamber with 5% CO_2_ at 37 °C with the medium exchanged every 3 days. When the cells reached 80–90% confluence, they were detached with HyClone^®^ HyQtase (GE, UT, USA) and replated at a ratio of 1:4. All the experiments used 3rd to 7th passages cells.

### MTT cell viability assay

hUCMSCs and HFLS-RA cells were plated in 96-well plates and then starved in a serum-free DMEM-LG medium for 16 h. After starvation, cells were stimulated with 100 ng/mL IL-1β for 24 h or with 25 μM, 50 μM, and 100 μM LFA-1-ICAM-1 inhibitor Lovastatin for an hour. After cytokine and inhibitor treatment, cells were incubated with 1 mg/mL MTT reagent (3-(4,5-Dimethylthiazol-2-yl)-2,5-diphenyltetrazolium bromide, SERVA, Heidelberg, Germany) in DMEM-LG medium for 4 h while protected from light. Aspirating the supernatant carefully, the formazan was dissolved with dimethyl sulfoxide (DMSO) for 2 h at 37 °C. The results were detected with Multimode micro-plate readers (Infinite 200, TECAN) at a wavelength of 545 nm.

### Pre-stimulating hUCMSCs or HFLS-RA cells with IL-1β

hUCMSCs or HFLS-RA were cultured in 10 cm petri dish til 80% confluence and then starved in serum-free DMEM-LG medium for 16 h. After starvation, cells were treated with 50 ng/mL, 100 ng/mL, and 200 ng/mL IL-1β for 24 h. Proteins were extracted immediately. The procedures are described in the Western blot passage.

### Adhesion assay

HFLS-RA were seeded in 96-well black plates. After forming a confluent monolayer, cells were starved in serum-free DMEM-LG medium for 16 h and stimulated with or without 100 ng/mL IL-1β for 24 h. At the same time, hUCMSCs were also cultured in 10 cm dishes with serum-free DMEM-LG medium for 16 h and with the same cytokine-stimulated condition of HFLS-RA. Before co-culturing, hUCMSCs were incubated with 2.5 μM Calcein AM (Tocris, UK) in serum-free DMEM-LG medium for 30 min at 37 °C protected from light, then washed with PBS 2 times and resuspended in DMEM-LG medium. hUCMSCs (5 × 10^3^/100 μL) were added into HFLS-RA wells with or without 50 μM Lovastatin for an hour adhesion at 37 °C protected from light. The non-adhesion cells in each well were washed out using PBS with Ca^2+^/Mg^2+^ 3 times. The results were detected by Multimode microplate readers (Infinite 200, TECAN) with a wavelength of 480 nm for excitation and 520 nm for emission.

### Fluorescence study of cell adhesion assay

HFLS-RA were seeded on 12 mm microscope cover glasses in 24-well culture plates. Then, the cells were starved in serum-free DMEM-Low Glucose medium (Gibco, NY, USA) for 16 h. After starvation, cells were stimulated with or without 100 ng/mL IL-1β for 24 h. Meanwhile, hUCMSCs were also starved in serum-free DMEM-LG medium for 16 h and with the same cytokine-stimulated condition of HFLS-RA. Before co-culturing, hUCMSCs were incubated with 6 μM Calcein AM (Tocris, UK) in serum-free DMEM-LG medium for 30 min at 37 °C protected from light. After incubation, cells were centrifuged and washed with PBS 2 times, then resuspended in DMEM-LG medium. hUCMSCs (5 × 10^4^/900 μL) were added into each HFLS-RA containing wells with or without 50 μM Lovastatin for an hour adhesion at 37 °C protected from light. After adhesion, the non-adhesion cells in each well were washed out using PBS with Ca^2+^/Mg^2+^ 3 times. Cells were fixed in 4% paraformaldehyde (Ferak, Berlin, Germany) at 4 °C overnight and stained with Hoechst 33258 (Sigma, MO, USA) at 1:5000 dilution after washing three times with PBS. Last of all, cells were mounted with Fluorescence Mounting Medium (Ibidi, Planegg, Germany). Images of cells were taken using Fluorescent Microscope (Leica DM6000B, Wetzlar, Germany) and the statistical results were counted with cell numbers in five random fields of view (FOV) at 10 × magnification.

### Western blot

Cells were washed with PBS and lysed by M-PER Mammalian Protein Extraction Reagent (Thermo, IL, USA) with 1% Halt Protease Inhibitor Cocktail (Thermo, IL, USA). The extraction was gently shaken for 3 min and centrifuged at 14,000*g* for 10 min, 4 °C. To isolate the cytosol proteins and membrane proteins, cells were lysed by Mem-PER™ Plus Membrane Protein Extraction Kit (Thermo, IL, USA). Cells were washed with PBS and detached by HyClone^®^ HyQtase (GE, UT, USA), and then centrifuged at 1000 rpm for 3 min to collect cell pellets. Cell pellet was washed with Cell Wash Solution and incubated with Permeabilization Buffer for 30 min at 4 °C on shaker, and then centrifuged at 16,000*g* for 15 min at 4 °C to collect the supernatant containing cytosol proteins. Solubilization Buffer was added to the pellet and mixed in by pipetting. The Mixture liquid was incubated for an hour at 4 °C and centrifuged at 16,000*g* for 15 min at 4 °C to collect the membrane proteins. Protein concentration was determined by Bio-Rad Protein Assay Dye Reagent (BIO-RAD, CA, USA) and multimode microplate readers (Infinite 200, TECAN). Protein samples were resolved using 10 or 15% sodium dodecyl sulfate–polyacrylamide gel electrophoresis (SDS-PAGE) and transferred to polyvinylidene fluoride (PVDF) membranes (BIO-RAD, CA, USA). After transferring, membranes were blocked with 5% Fish Gelatin Blocking Buffer (AMRESCO, OH, USA) in tris-buffered saline with tween-20 (TBST) for an hour at room temperature. After blocking, membranes were incubated with primary antibody LFA-1(GeneTex, CA, USA) with 1:1000 dilution, pan-cadherin antibody (Abcam, Cambridge, UK) diluted at 1:2000, TRAIL R1 (Novus, St. Charles, USA) with 1:2000 dilution, DR5 antibody (Abcam, Cambridge, UK) with 1:1000 dilution, ICAM-1 antibody (Abcam, Cambridge, UK) with 1:2000 dilution, TRAIL antibody (Abcam, Cambridge, UK) with 1:2000 dilution or beta-actin antibody (GeneTex, CA, USA) with 1:5000 dilution in 5% Fish Gelatin Blocking Buffer TBST at 4 °C overnight. The membranes were washed with TBST three times for 5 min each, then incubated with horseradish peroxidase-conjugated (HRP) Mouse or Rabbit IgG secondary 20 antibodies (GeneTex, CA, USA) for an hour at room temperature. The membranes were washed with TBST three times for 5 min each. The proteins were visualized using the LAS-4000 Luminescence Imaging System (GE, CT, USA) with enhanced chemiluminescence substrate (ECL) (PerkinElmer, MA, USA).

### Cell immunofluorescence and images

hUCMSCs and HFLS-RA were seeded on 12 mm microscope cover glasses in 24-well culture plates. Then, the cells were starved in serum-free DMEM-LG for 16 h. After starvation, cells were stimulated with IL-1β in different concentrations or different durations. For different concentrations of IL-1β, cells were treated with 50 ng/mL, 100 ng/mL, 200 ng/mL IL-1β for 24 h. For difference in duration, cells were treated with 100 ng/mL IL-1β for 6, 16, 24, or 48 h. After cytokine stimulation, cells were fixed in 4% paraformaldehyde (Ferak, Berlin, Germany) at 4 °C overnight, and were permeabilized with 0.1% Triton X-100 (Sigma, MO, USA) in PBS for 10 min. Next, cells were blocked with 2% bovine serum albumin (BSA) (Sigma, MO, USA) for 30 min, and then incubated with primary antibody TRAIL R1 (Novus, St. Charles, USA) with 1:200 dilution, DR5 antibody (Abcam, Cambridge, UK) with 1:100 dilution, ICAM-1 antibody (Abcam, Cambridge, UK) with 1:200 dilution, or TRAIL antibody (Abcam, Cambridge, UK) with 1:200 dilution at 4 °C overnight. After washing three times with PBS, cells were incubated for an hour at room temperature with Alexa Fluor 488-conjugated AffiniPure Rabbit Anti-Mouse or Goat Anti-rabbit secondary antibody (Jackson ImmunoResearch Laboratories, PA, USA) for a dilution of 1:100 or 1:200 depending on each primary antibody. Then, cells were stained with Hoechst 33258 (Sigma, MO, USA) at 1:5000 dilution after washing three times with PBS. Last of all, cells were mounted with Fluorescence Mounting Medium (Ibidi, Planegg, Germany). Images of cells were captured using Fluorescent Microscope (Leica DM6000B, Wetzlar, Germany) or Laser Confocal Microscope (LSM880, Oberkochen, Germany).

### LFA-1 siRNA oligonucleotides and negative control siRNA transfection

LFA-1 Silencer Select predesigned siRNA (s534808 and s229716, Ambion, Austin, USA) and Silencer Select negative control no. 1 (Ambion, Austin, USA) were used to downregulate LFA-1 expression in cells. When cells were cultured in 10 cm dish until 80% confluence, 5 nM LFA-1-specific siRNA or negative control siRNA and lipofectamine^®^ RNAiMAX transfection reagent dilute in Opti-MEM^®^ Medium (Invitrogen, CA, USA) was prepared following by the manufacturer’s instruction. Then cells were cultured in the 1:1 mixture of dilute siRNA and dilute lipofectamine^®^ RNAiMAX for 24 h.

### Direct co-culture of hUCMSCs and HFLS-RA

hUCMSCs were seeded in 6-wells and HFLS-RA were seeded in 6-wells or cultured on 12 mm microscope cover glasses in 24-wells culture plate. Both cells were stimulated with/without 100 ng/mL IL-1β for 24 h. Then 5 × 10^5^ of hUCMSCs were added to 6 wells and 5 × 10^4^ hUCMSCs were added to 24 wells of HFLS-RA containing wells for 24 h. For Western blot, cells were washed with PBS, then the cells were lysed using M-PER Mammalian Protein Extraction Reagent (Thermo, IL, USA) with 1% Halt Protease Inhibitor Cocktail (Thermo, IL, USA). Extractions were vortexed for 1 min and centrifuged at 14,000*g* for 10 min at 4 °C.

### Caspase-8, -9, and -3 detection in directly co-culture HFLS-RA and hUCMSCs

HFLS-RA were cultured in 24-wells and hUCMSCs were cultured in 10 cm dishes. For cytokine stimulation, hUCMSCs and HFLS-RA were stimulated with 100 ng/mL IL-1β for 24 h. After cytokine stimulation, hUCMSCs were labeled with 5 μM CellTracker Orange (Life technology, NY, USA) for 30 min at 37 °C, protected from light. Cells were centrifuged at 1500 rpm for 5 min and washed 3 times with PBS, protected from light. Finishing the label steps, 5 × 10^4^ of hUCMSCs cells were added to HFLS-RA containing 24-wells. After 24 h direct co-culture, cells were fixed in 4% paraformaldehyde (Ferak, Berlin, Germany) at 4 °C overnight, and were permeabilized with 0.1% Triton X-100 (Sigma, MO, USA) in PBS for 10 min. Next, cells were blocked with 2% BSA (Sigma, MO, USA) for 30 min, and then incubated with primary antibody of caspase-8, caspase-9 or caspase-3 (GeneTex, CA, USA) with 1:200 dilution, at 4 °C overnight. After washing three times with PBS, cells were incubated an hour at room temperature with Alexa Fluor 488-conjugated AffiniPure Goat Anti-rabbit secondary antibody (Jackson ImmunoResearch Laboratories, PA, USA) for a dilution of 1:200. Then, cells were stained with Hoechst 33258 (Sigma, MO, USA) at 1:5000 dilution after washing three times with PBS. Last of all, cells were mounted with Fluorescence Mounting Medium (Ibidi, Planegg, Germany). Images of cells were obtained using Fluorescent Microscope (Leica DM6000B, Wetzlar, Germany).

### Annexin V/PI detection by immunofluorescence study

HFLS-RA cells were seeded on 12 mm microscope cover glasses and cultured in a 24-well plate, and hUCMSCs were cultured in 10 cm dishes. For cytokine stimulation, hUCMSCs and HFLS-RA were stimulated with 100 ng/mL IL-1β for 24 h. After cytokine stimulation, hUCMSCs were labeled with 5 μM CellTracker Orange (Life technology, NY, USA) for 30 min at 37 °C, protected from light. After labeling, cells were centrifuged at 1500 rpm for 5 min and washed 3 times with PBS, then centrifuged at 1500 rpm protected from light. Finishing the labeling steps, 5 × 10^4^ of hUCMSCs cells were added to HFLS-RA containing 24-wells. After 24 h of direct co-culture, cells were stained with Annexin V-FITC Apoptosis Detection Kit (Strong Biotech Corporation, Taipei, Taiwan). According to the manual guide, the procedures are briefly described below. 2 μL of Annexin V-FITC was diluted in 100 μL Binding Buffer and 2 μL PI added for each assay as the preparation of staining solution. The cover glasses were removed and 100 μL staining solution was applied to each sample for 15 min incubation at room temperature. After incubation, samples were immediately examined and captured by Fluorescent Microscope (Leica DM6000B, Wetzlar, Germany).

### Animal experiment and the induce of collagen-induced arthritis

All experimental were approved by the Institutional Animal Care and Use Committee (IACUC) of National Yang Ming Chiao Tung University (on April 20, 2018) and were conducted in accordance with ethical regulations (Approval no. 1070410). Reporting of animal experiments follows recommendations in the ARRIVE guidelines. The mice were maintained in the Laboratory Animal Center of National Yang Ming Chiao Tung University in a 12 h dark/12 h light cycle with abundant food and water. Eight weeks old DBA/1J mice were used for all experiments in this study (n = 60). If the animal died during the experiment, it was excluded from the collection of data.

All the experiments adhered to the “Protocol for the Successful Induction of Collagen-Induced Arthritis (CIA) in Mice” (Chondrex, Inc.) Eight weeks old DBA/1J mice were injected intradermally (i.d) with 100 μg of bovine type II collagen (CII) (Chondrex, Redmond, WA, USA) emulsified with Complete Freund’s Adjuvant (CFA) (Chondrex, Redmond, WA, USA) at the base of the tail. For a booster injection, Incomplete Freund’s adjuvant (IFA) containing 100 μg of CII was administered on the day 21. Arthritis would develop 28–35 days after the first injection. On day 36, 1 × 10^6^ hUCMSCs in 100 μL PBS were administered via tail vein. The Sham group was injected with the same volume of PBS as the inducing agent and hUCMSCs. Mice were sacrificed by cervical dislocation after isoflurane anesthesia on day 76 after starting CIA induction.

### Therapeutic efficacy evaluation of hUCMSCs on CIA mouse model

The severity of RA symptom was evaluated with paw thickness using a digital caliper and body weight was measured with an electronic weigher every 10 days for four periods. According to the “Mouse Arthritis Scoring System” (Chondrex, Inc), clinical scores were judged on the redness and swelling of front and hind paws of each mouse. Three joint types were observed for scoring: the interphalangeal joint, the metacarpophalangeal joint, and the carpal and tarsal joint. The score is defined as follow: score 0 = normal, score 1 = one joint type has redness and swelling, score 2 = two joint types have redness and swelling, score 3 = all three joints have redness and swelling, score 4 = symptoms of the entire paw were maximally severe and the anatomic appearance was hard to distinguish. The total score was obtained from 4 paws, so the maximum score was 16 for each mouse.

### Hematoxylin and eosin (H&E) stain

After sacrificing, the front and hind limbs were removed for histopathological examination. The tissues were fixed, decalcified and embedded in paraffin, then sectioned into 5 μm slides. To describe briefly, the slides were deparaffinized and rehydrated before staining with hematoxylin and eosin (H&E). The results were observed and photographed by microscope.

### TUNNEL assay

After sacrificing, the hind legs were removed for histopathological examination. The tissues were fixed, decalcified and embedded with paraffin, then sectioned into 5 μm slides. The slides were deparaffinized and rehydrated, then processed following the manufacturer’s instructions of TUNEL Assay Kit—HRP-DAB (Abcam, Cambridge, UK). The results were observed and photographed by microscope.

### ^18^F-FDG microPET/MRI imaging

^18^F-FDG was used to track the inflammatory response. The response was evaluated every 10 days after hUCMSCs administration. The mice were anesthetized using 1–3% isoflurane and intravenously injected with 7.4 MBq/0.2 mL ^18^F-FDG for an hour. All images were acquired for 10 min by Bruker 7T PET/MR after injection. The regions of interest (ROI) were selected and the accumulation of ^18^F-FDG was measured using Amide’s Medical Imaging Data Examiner (AMIDE) software. Standardized uptake value (SUV) was calculated from the palm, knee and paw.

### Statistical analysis

Quantitation data was analyzed by Student’s t-test and one-way ANOVA. P values < 0.05 were considered as statistically significant. All statistical analysis was performed with Prism 5 software.

### Ethics approval and consent to participate

All experimental protocols were approved on April 20, 2018 by the Institutional Animal Care and Use Committee (IACUC) of National Yang Ming Chiao Tung University and were conducted in accordance with ethical regulations (Approval no. 1070410). The title of the approved project is “Effects of Human Umbilical Cord Wharton’s Jelly-derived Mesenchymal Stem Cells on Fibroblast-like Synoviocytes from Rheumatoid Arthritis”.

### Supplementary Information

Supplementary Figures.

## Data Availability

All data needed to evaluate the conclusions in the paper are present in the paper.
