# Supplementary material for: IL-1β stimulated human umbilical cord mesenchymal stem cells ameliorate rheumatoid arthritis via inducing apoptosis of fibroblast-like synoviocytes
Source: Sci Rep. 2023 Sep 15;13:15344. doi: 10.1038/s41598-023-42585-1 (PMC10504325; doi:10.1038/s41598-023-42585-1)

IL-1β Stimulated Human Umbilical Cord Mesenchymal Stem Cells Ameliorate Rheumatoid Arthritis via Inducing Apoptosis of Fibroblast-like Synoviocytes

Yun-Hsuan Chiu[1], Ya-Han Liang[1], Jeng-Jong Hwang[2] and Hwai-Shi Wang[1,]*

Supplementary Figure 1. Time-dependent effect of IL-1β stimulation on ICAM-1 expression in HFLS-RA cells.

(a) Immunofluorescence study of ICAM-1 expression with 100 ng/ml IL-1β stimulation in 6, 16, 24, 48 hours. Green: ICAM-1, blue: Hoechst 33258 (nucleus), scale bar: 100 μm. White blocks chosen are magnified in the column below. (b) Quantitative fluorescence intensity results of (A) analyzed by Image J. The data represent mean ± SD (n=3) (**P*<0.05, ***P*<0.01, ***P<0.001).

(a)

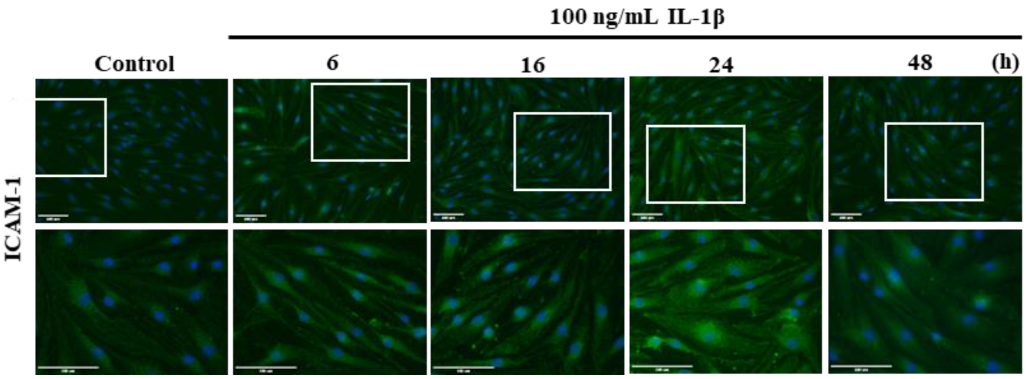

(b)

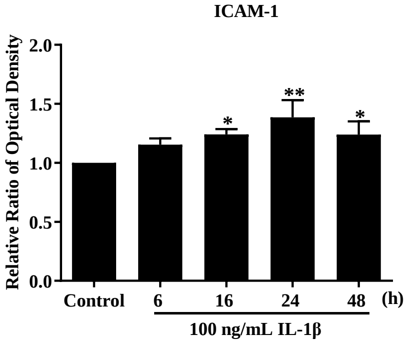

Supplementary Figure 2. The effect of IL-1β on hUCMSCs and HFLS-RA cells by MTT assay.

(a) The cell viability of hUCMSCs and (b) HFLS-RA cells treated with 100 ng/ml IL-1β, and different concentrations of LFA-1 antagonist-Lovastatin. DMSO was the solvent of Lovastatin, also investigated in MTT assay. The data represent mean ± SD (n=3) (**P*<0.05).

(a)

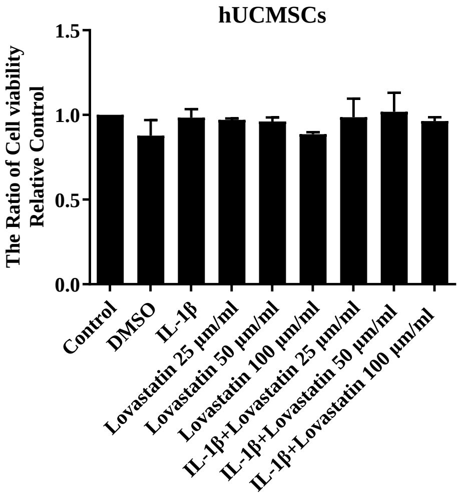

(b)

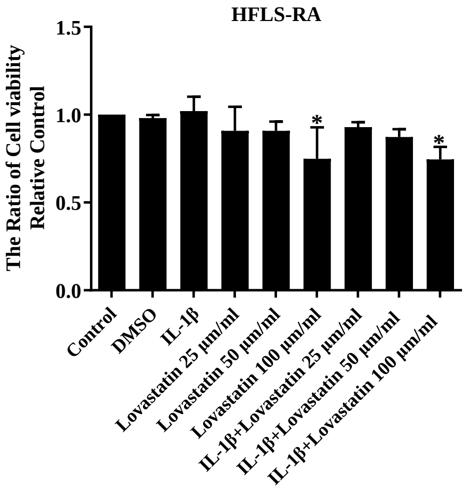

Supplementary Figure 3. Effects of IL-1β on DR4 and DR5 expression in HFLS-RA cells.

(a) Immunofluorescence study of DR4 expression with 100 ng/ml IL-1β stimulation in 6, 16, 24, 48 hours. Green: DR4, blue: Hoechst 33258 (nucleus), scale bar: 100 μm. White blocks chosen are magnified in the column below. (b) Quantitative fluorescence intensity results of (a) analyzed by ImageJ. (c) Immunofluorescence study of DR5 expression with 100 ng/ml IL-1β stimulation in 6, 16, 24, 48 hours. Green: DR5, blue: Hoechst 33258 (nucleus), scale bar: 100 μm. White blocks chosen are magnified in the column below. (b) Quantitative fluorescence intensity results of (c) analyzed by ImageJ. The data represent mean ± SD (n=3) (*P<0.05, **P<0.01, ***P<0.001).

(a)

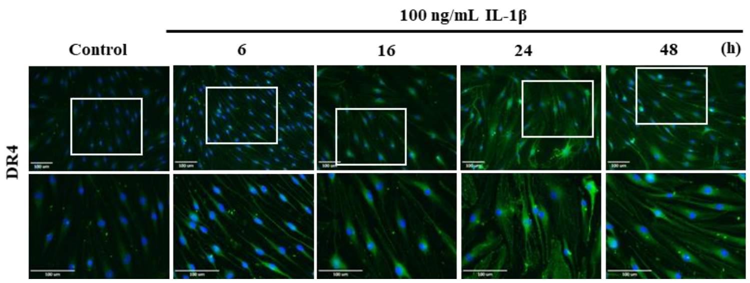

(b)

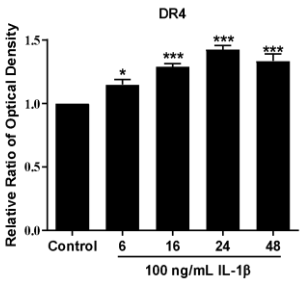

(c)

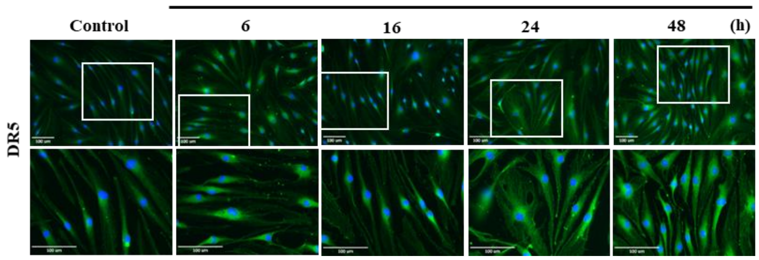

(d)

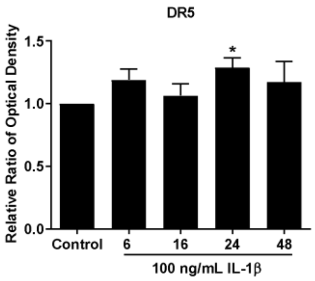

Supplementary Figure 4. The therapeutic efficacy of hUCMSCs by evaluating the exterior appearance.

(a) Statistical results of body weight after hUCMSCs administration. (b) After hUCMSCs administration, mice were anesthetized to record the exterior appearance in order to evaluate therapeutic efficacy. On day 40, with hUCMSCs and IL-1β stimulated hUCMSCs administration, the symptoms of redness and swelling were significantly improved. Arrow head: the redness and swelling of the front and hind paws. The data represent mean ± SD (n=7 per group).

(a)

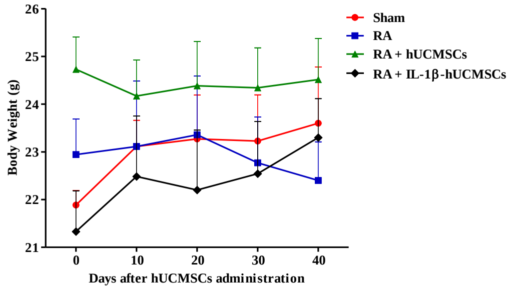

(b)

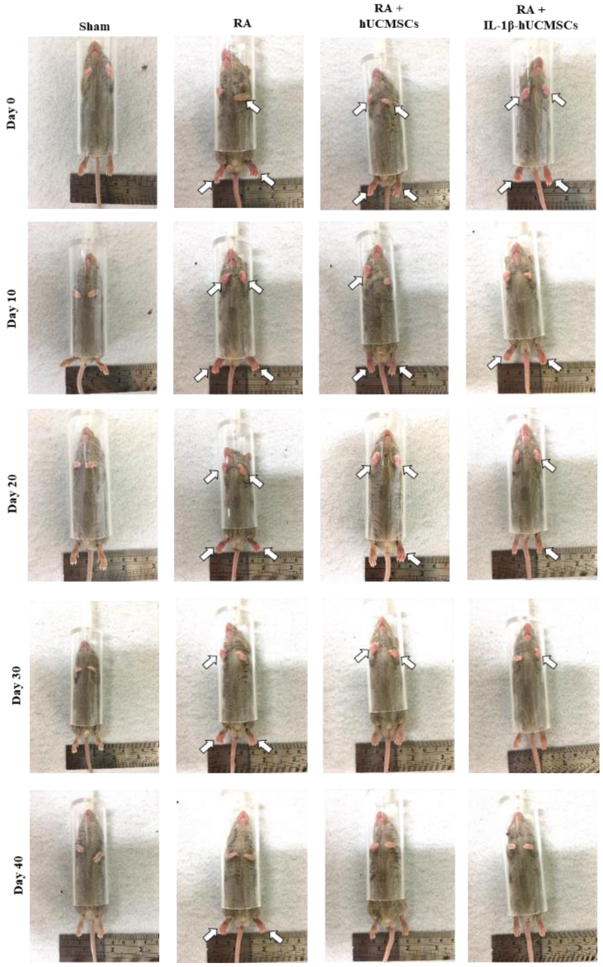

Supplementary Figure 5. The group of RA+ hUCMSCs and RA+ IL-1β-hUCMSCs on Day 20 in Fig. 8b. Arrow: the apoptotic fibroblast-like cells in the apoptosis region.

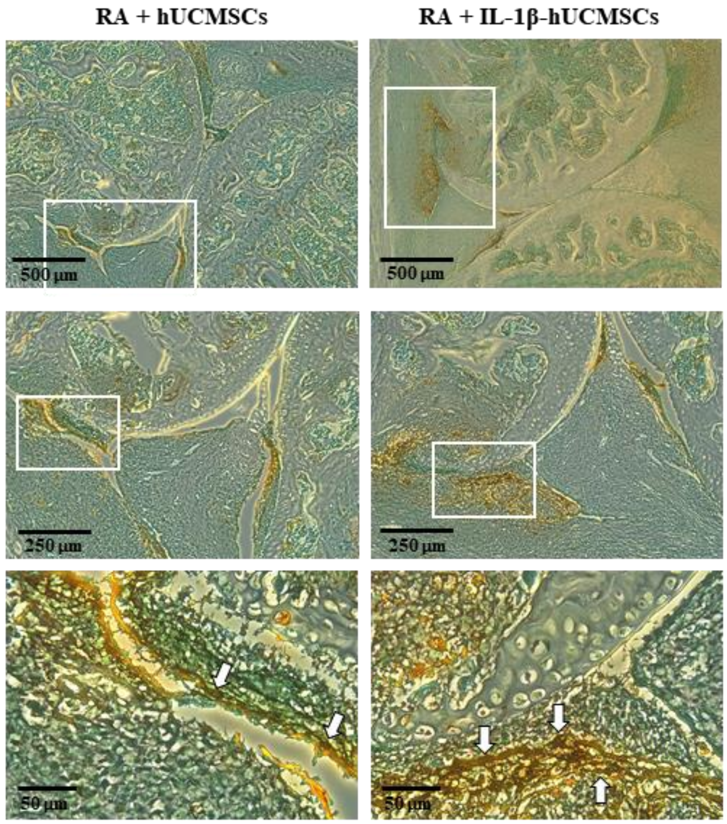

Supplementary Figure 6. Full- length Western blots.

Figure 1 (a)

Lane group：Control/ 50/ 100/ 200 IL-1β ng/mL MW(kDa)

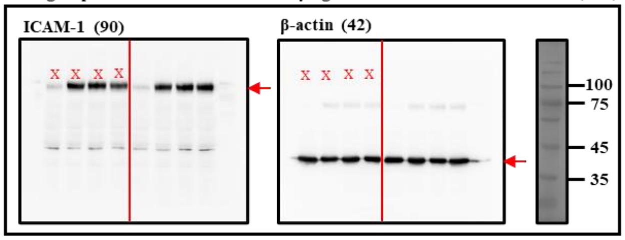

Figure 2 (d)

lane group: hUCMSCs /IL-1β-hUCMSCs /LFA-1 siRNA / LFA-1 siRNA+ IL-1β-hUCMSCs MW(kDa)

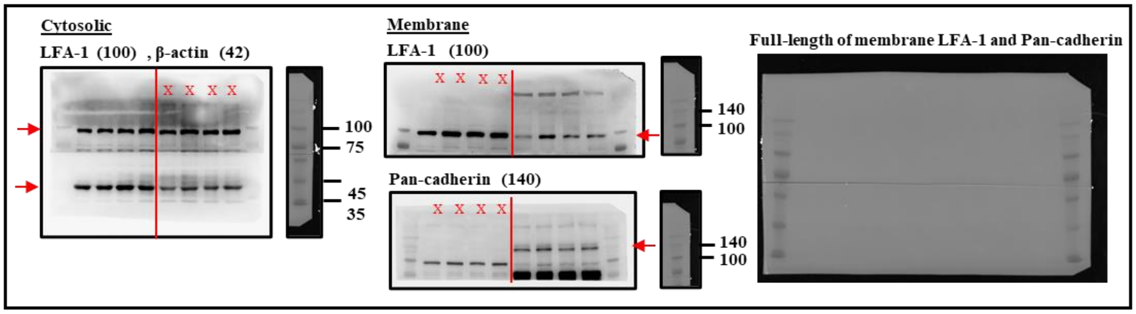

Figure 3 (a)

lane group: control /50/100/200 IL-1β ng/mL MW(kDa)

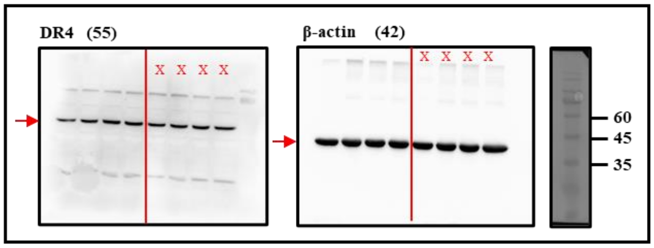

Figure 3 (c)

lane group: control /50/100/200 IL-1β ng/mL MW(kDa)

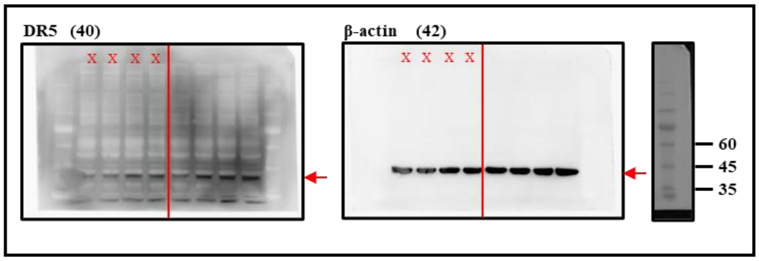

Figure 4 (a)

lane group: control /50/100/200 IL-1β ng/mL MW(kDa)

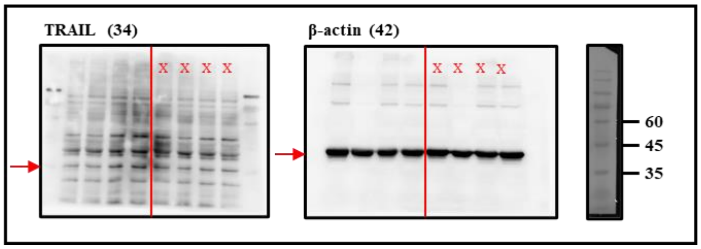

Figure 5 (b)
lane group: control/ hUCMSCs /IL-1β-hUCMSCs/
(IL-1β100ng/mL) control/ (IL-1β100ng/mL) hUCMSCs /(IL-1β100ng/mL) IL-1β-hUCMSCs MW(kDa)

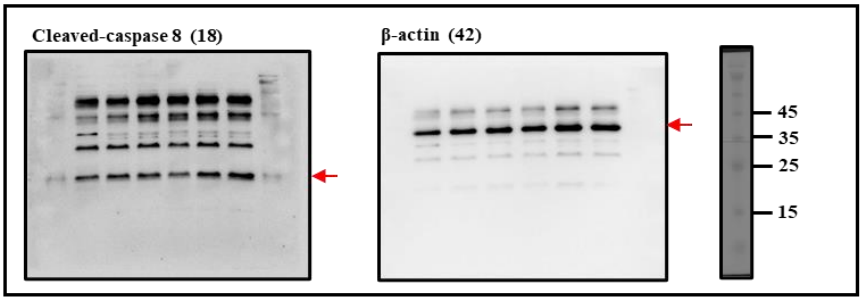

Figure 5 (e)
lane group: control/ hUCMSCs /IL-1β-hUCMSCs/
(IL-1β100ng/mL) control/ (IL-1β100ng/mL) hUCMSCs / (IL-1β100ng/mL) IL-1β-hUCMSCs MW(kDa)

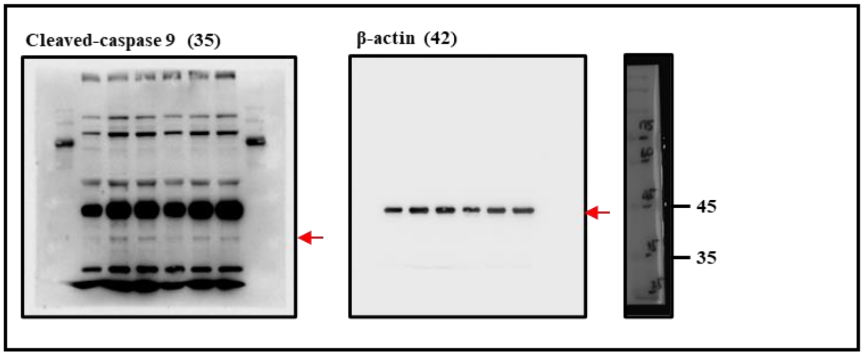

Figure 5 (h) MW(kDa)
lane group: control/ hUCMSCs /IL-1β-hUCMSCs/
(IL-1β100ng/mL) control/ (IL-1β100ng/mL) hUCMSCs / (IL-1β100ng/mL) IL-1β-hUCMSCs MW(kDa)

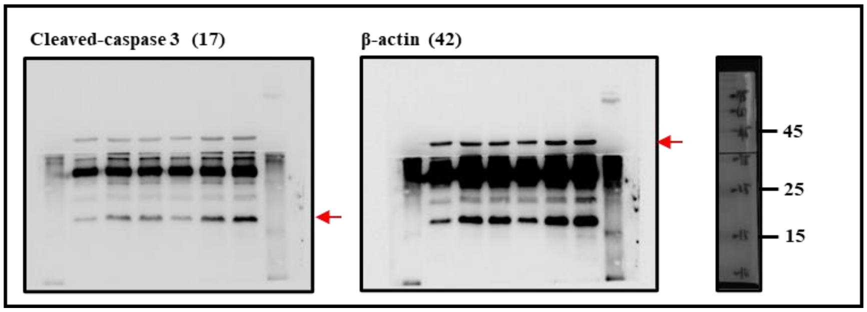

Supplement: Supplementary file 1 — Supplementary Figures. [file 41598_2023_42585_MOESM1_ESM.pdf]
